# Thyroid hormone modulates hyperoxic neonatal lung injury and mitochondrial function

**DOI:** 10.1172/jci.insight.160697

**Published:** 2023-04-24

**Authors:** Bianca M. Vamesu, Teodora Nicola, Rui Li, Snehashis Hazra, Sadis Matalon, Naftali Kaminski, Namasivayam Ambalavanan, Jegen Kandasamy

**Affiliations:** 1 Department of Pediatrics, School of Medicine, University of Alabama at Birmingham, Birmingham, Alabama, USA.; 2 Department of Pediatrics, College of Medicine, University of South Alabama, Mobile, Alabama, USA.; 3 Division of Molecular and Translational Biomedicine, Department of Anesthesiology and Perioperative Medicine, and Pulmonary Injury and Repair Center, School of Medicine, University of Alabama at Birmingham, Birmingham, Alabama, USA.; 4 Section of Pulmonary, Critical Care and Sleep Medicine, Department of Internal Medicine, Yale School of Medicine, New Haven, Connecticut, USA.; 5 Department of Pathology, School of Medicine, University of Alabama at Birmingham, Birmingham, Alabama, USA.

**Keywords:** Pulmonology, Human stem cells, Mitochondria

## Abstract

Mitochondrial dysfunction at birth predicts bronchopulmonary dysplasia (BPD) in extremely low–birth weight (ELBW) infants. Recently, nebulized thyroid hormone (TH), given as triiodothyronine (T3), was noted to decrease pulmonary fibrosis in adult animals through improved mitochondrial function. In this study, we tested the hypothesis that TH may have similar effects on hyperoxia-induced neonatal lung injury and mitochondrial dysfunction by testing whether i.n. T3 decreases neonatal hyperoxic lung injury in newborn mice; whether T3 improves mitochondrial function in lung homogenates, neonatal murine lung fibroblasts (NMLFs), and umbilical cord–derived mesenchymal stem cells (UC-MSCs) obtained from ELBW infants; and whether neonatal hypothyroxinemia is associated with BPD in ELBW infants. We found that inhaled T3 (given i.n.) attenuated hyperoxia-induced lung injury and mitochondrial dysfunction in newborn mice. T3 also reduced bioenergetic deficits in UC-MSCs obtained from both infants with no or mild BPD and those with moderate to severe BPD. T3 also increased the content of peroxisome proliferator–activated receptor γ coactivator 1α in lung homogenates of mice exposed to hyperoxia as well as mitochondrial potential in both NMLFs and UC-MSCs. ELBW infants who died or developed moderate to severe BPD had lower total T4 (TT4) compared with survivors with no or mild BPD. In conclusion, TH signaling and function may play a critical role in neonatal lung injury, and inhaled T3 supplementation may be useful as a therapeutic strategy for BPD.

## Graphical abstract

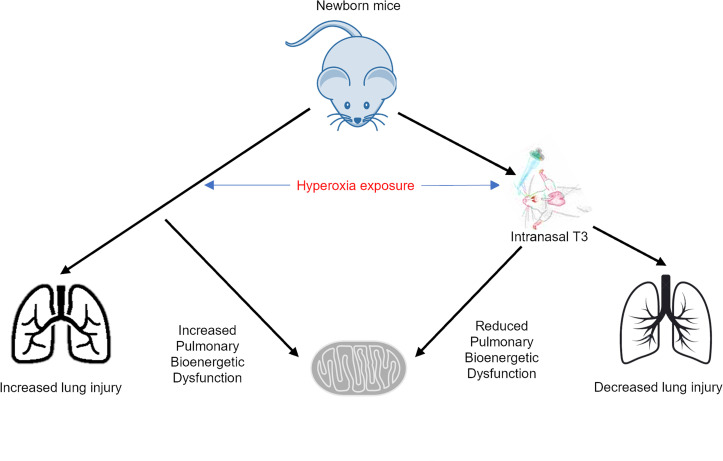

## Introduction

Extremely low–birth weight (ELBW; <1,000 g BW) infants frequently require prolonged oxygen supplementation to compensate for their pulmonary immaturity. Oxidative stress secondary to such hyperoxia exposure is a significant cause of impaired lung development and increased pulmonary fibrosis that results in bronchopulmonary dysplasia (BPD), which is a significant morbidity ELBW infants experience that continues to remain highly prevalent despite advances in their medical care (1–3). Therefore, there is an urgent need for exploration of novel mechanisms that mediate neonatal oxidative stress and lung development. Mitochondria have recently been found to be major sources as well as targets of oxidative stress, and mitochondrial dysfunction has recently been shown to increase type 2 alveolar epithelial cell (AT2) apoptosis and risk for disorders such as pulmonary fibrosis, asthma, and bronchitis (4, 5). We and others have also found that mitochondrial bioenergetic and redox dysfunction is associated with hyperoxic lung injury in newborn mice as well as increased risk for BPD in ELBW infants (6–9).

Thyroid hormones (THs), including thyroxine (T4) and triiodothyronine (T3), are involved in the regulation of fetal lung development (10). THs are also known to be key regulators of mitochondrial transcriptional pathways and biogenesis (11, 12), and hyperoxia exposure decreases serum T4 levels in mice (13). Therefore, decreasing oxidative stress–induced mitochondrial dysfunction through TH supplementation could be a useful strategy for decreasing neonatal lung injury and BPD. However, the limited evidence currently available suggests that systemic TH administration may offer only minimal respiratory benefits for preterm infants with respiratory distress syndrome or BPD (14, 15). In this context, aerosolized T3, delivered directly into lungs of adult mice, has been found to decrease airway fibrosis through improved AT2 mitochondrial function (16, 17). Whether such localized TH delivery into lungs could similarly improve neonatal hyperoxic lung injury and decrease BPD risk in ELBW infants remains unknown. The effects of TH supplementation on neonatal mitochondrial dysfunction caused by oxidative stress have not been examined previously. Finally, state newborn screens (NBS) routinely measure total T4 (TT4) in ELBW infants to screen for hypothyroidism at various time points during the first month of life, but the association between these NBS TT4 measurements and BPD risk has not been evaluated in ELBW infant cohorts from the United States.

Consequently, this study tested the hypothesis that TH function and neonatal hypothyroxinemia are associated with neonatal hyperoxic lung injury, BPD risk, and mitochondrial dysfunction. First, we tested whether i.n.-instilled T3 modifies neonatal lung development and whole lung homogenate mitochondrial dysfunction in an established model of murine hyperoxia-induced BPD. Next, we examined the effects of T3 supplementation on mitochondrial function in AT2s and neonatal murine lung fibroblasts (NMLFs) as well as mesenchymal stem cells isolated from umbilical cords (UC-MSCs) obtained from ELBW infants at their birth. Finally, we also evaluated the association between NBS TT4 levels and BPD risk in ELBW infants admitted to the neonatal intensive care unit of the University of Alabama at Birmingham (UAB).

## Results

### I.n. T3 improves lung function and structure in newborn mice exposed to hyperoxia.

C57BL/6 newborn mice were assigned to the air vehicle, air T3, hyperoxia vehicle, or hyperoxia T3 group at P4–P14. A minimum of 6 mice per group were used for all experiments performed. Mice exposed to the experimental interventions including hyperoxia and i.n. T3 had similar survival rates, weights, and lengths compared to the controls. However, hyperoxia exposure was noted to decrease serum T4 levels in mice (Supplemental Figure 1; supplemental material available online with this article; https://doi.org/10.1172/jci.insight.160697DS1).

**Figure 1:**
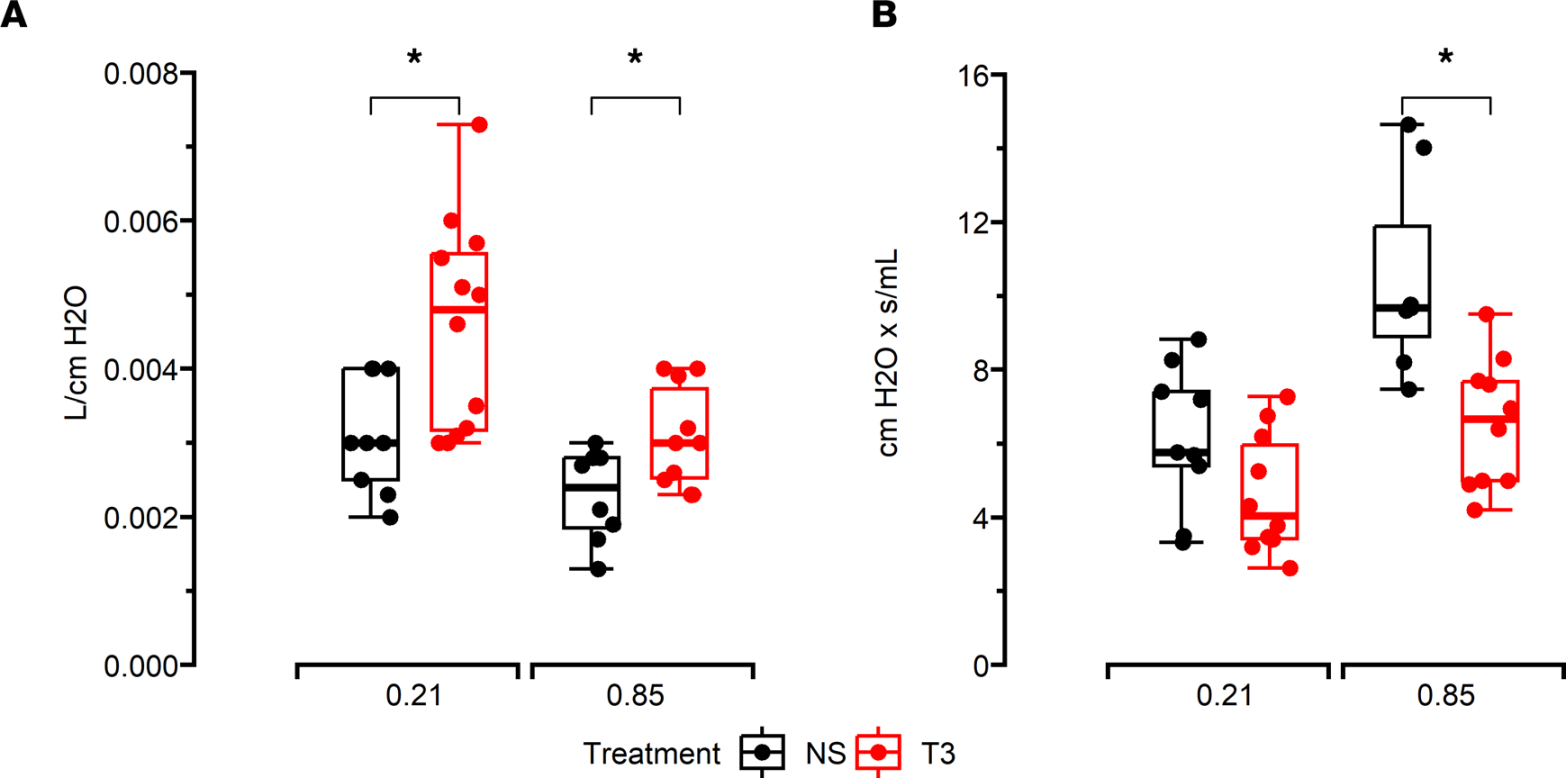
Lung function in mice. (**A**) Pulmonary compliance and (**B**) airway resistance in lungs from newborn mice exposed to air, air and T3, hyperoxia, and hyperoxia and T3 measured using Flexivent. T3 improved both compliance and resistance in hyperoxia-exposed mice and only compliance in mice exposed to air (normoxia). *N* = minimum of 6 mice/group. All data were analyzed by 2-way ANOVA or Kruskal-Wallis tests, followed by post hoc analyses. Box represents median/IQR; whiskers represent maximum and minimum values. *P < 0.05.

Lung function was assessed in mice at P14 by measuring pulmonary compliance and airway resistance through the forced oscillation technique using a FlexiVent and pressure-volume loops (Supplemental Figure 2). Hyperoxia vehicle–treated mice had lower pulmonary compliance (mean ± SEM; 0.002 ± 0.001 vs. 0.003 ± 0.001 L/cm H_2_O; *P* < 0.05) and increased airway resistance (11 ± 1 vs. 6 ± 1 cm H_2_O × s/mL; *P* < 0.05) compared with mice from the air vehicle group. Hyperoxia T3–treated mice had increased pulmonary compliance (mean ± SEM; 0.003 ± 0.001 vs. 0.002 ± 0.001 L/cm H_2_O; *P* < 0.05) and lower airway resistance (7 ± 2 vs. 11 ± 3 cm H_2_O × s/mL; *P* < 0.05) compared with mice from the hyperoxia vehicle group. Additionally, normoxia T3–treated mice were noted to have increased pulmonary compliance but similar airway resistance when compared with normoxia vehicle mice (Figure 1). These results indicate that while i.n. T3 improved pulmonary compliance during normoxia as well, it improved hyperoxia-induced changes in lung elasticity and airway reactivity to a greater degree in newborn mice.

**Figure 2:**
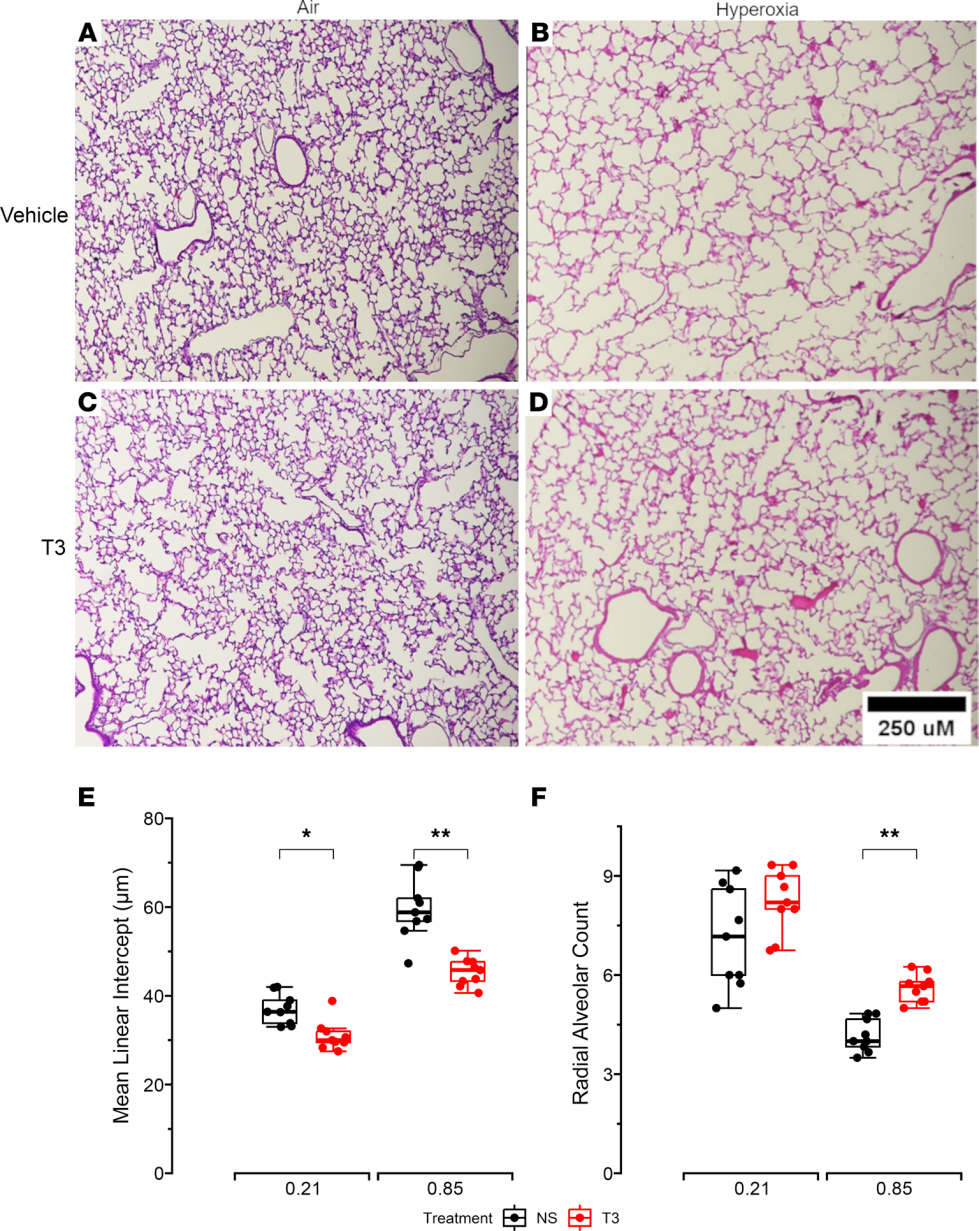
Alveolar development and lung morphometry in 14-day-old mice. (**A**–**D**) Representative photographs of H&E-stained lung sections from mice that received vehicle (normal saline) or T3 while being exposed to air or hyperoxia: (**A**) vehicle air, (**B**) vehicle hyperoxia, (**C**) T3 air, and (**D**) T3 hyperoxia. (**E**) MLI. (**F**) RAC. *N* = minimum of 6 mice/group. All data were analyzed by 2-way ANOVA or Kruskal-Wallis tests, followed by post hoc analyses. Box represents median/IQR; whiskers represent maximum and minimum values. *P < 0.05; **P < 0.005.

H&E-stained lung sections were examined to study morphology. Lung sections from the hyperoxia vehicle group had a larger mean linear intercept (MLI) and lower radial alveolar count (RAC) compared with mice in the air vehicle group, indicating that, as expected, hyperoxia disrupted alveolarization in our model. I.n. T3 supplementation caused a reduction in MLI and increased RAC in lung sections of mice exposed to hyperoxia when compared with the control group, indicating that i.n. T3 supplementation attenuates the disrupted alveolarization caused by hyperoxia in newborn mice. Although decreased MLI was also noted in the lung sections of mice in the normoxia T3 group versus the normoxia vehicle group, T3 had no effects on RAC in lung sections of mice exposed to normoxia, indicating that T3 helped improve lung structure in hyperoxia-exposed mice to a greater degree (Figure 2).

### I.n. T3 improves lung mitochondrial function in newborn mice exposed to hyperoxia.

**Figure 3:**
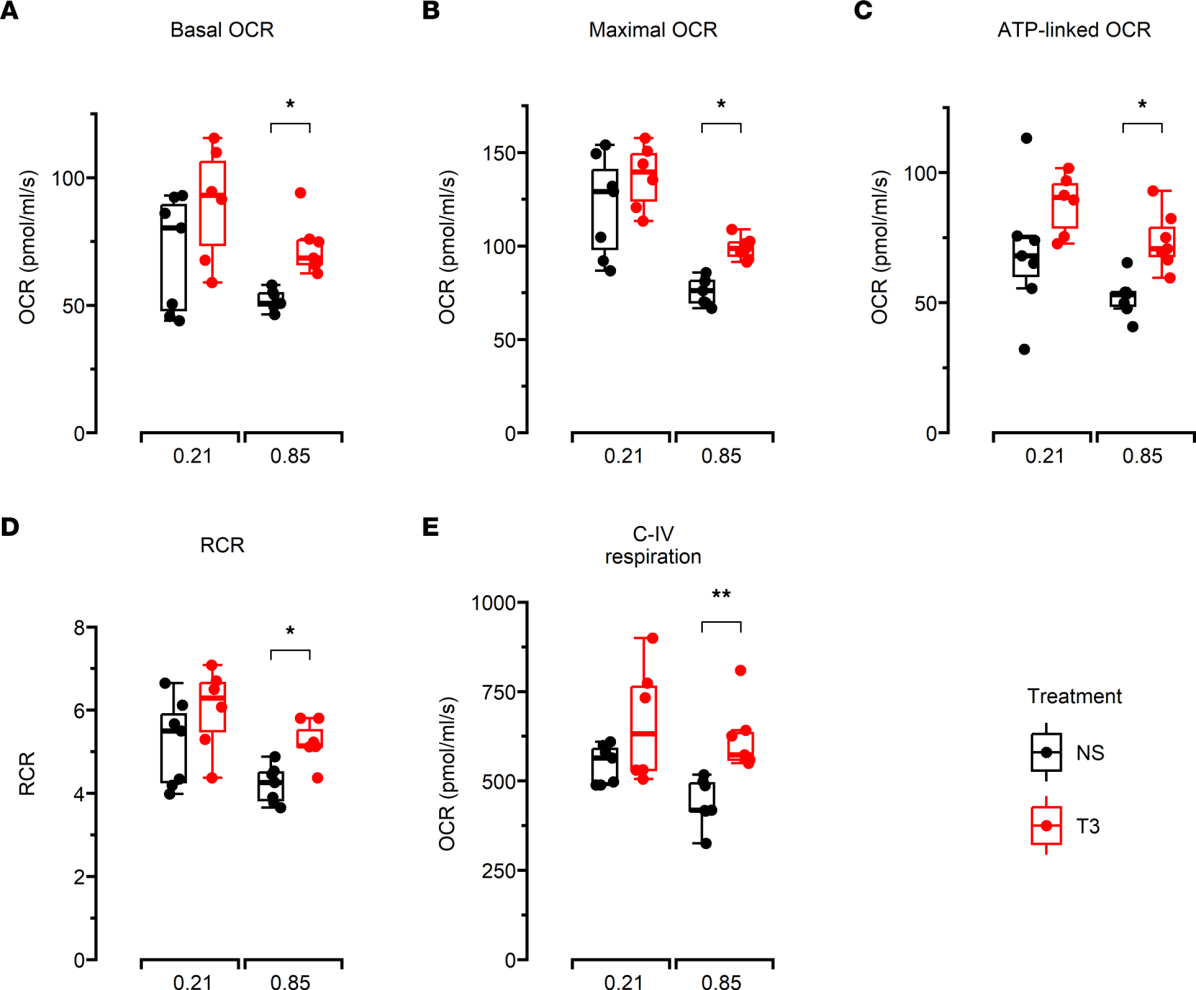
Bioenergetic function and electron transport chain complex activities of lung mitochondria. A total of 300 μg mitochondria per sample isolated from lung homogenates of 14-day-old mice that received vehicle (normal saline) or T3 while being exposed to air or hyperoxia used to measure OCR in the presence of various substrates and mitochondrial effectors. (**A**) Basal OCR. (**B**) Maximal OCR. (**C**) ATP-linked OCR. (**D**) RCR, which is a measure of mitochondrial oxidative phosphorylation efficiency. (**E**) Complex IV (C-IV) activity. *N* = minimum of 6 mice/group. All data were analyzed by 2-way ANOVA or Kruskal-Wallis tests, followed by post hoc analyses. Box represents median/IQR; whiskers represent maximum and minimum values. *P < 0.05; **P < 0.005. NS, normal saline.

The basal oxygen consumption rate (OCR) of cell-free mitochondria isolated from lung homogenates measured in the presence of excess substrate and the absence of any mitochondrial effector, maximal OCR measured after addition of the protonophore carbonyl cyanide-*p*-trifluoromethoxyphenylhydrazone (FCCP), and ATP-linked OCR measured after addition of the ATP synthase inhibitor oligomycin were all lower in the hyperoxia vehicle group compared with the air vehicle group. Such hyperoxia-induced pulmonary mitochondrial bioenergetic dysfunction was noted to be markedly attenuated in hyperoxia-exposed mice supplemented with T3 compared with hyperoxia-exposed controls. The respiratory control ratio (RCR), which indicates mitochondrial capacity for substrate oxidation and ATP turnover, was similar between the air vehicle, air T3, and hyperoxia T3 groups but lower in the hyperoxia vehicle group (Figure 3, A–D). Basal OCR and ATP-linked OCR were similar between saline-treated normoxic and hyperoxic mice, whereas maximal OCR was noted to be significantly lower in the hyperoxic mice. These observations were also noted in mice treated with T3 (Supplemental Figure 3).

Electron transport chain (ETC) respiratory activities in mouse lung homogenates mediated by complex I (C-I) and complex IV (C-IV) were also assessed using succinate, N, N, N′, N′-tetramethyl-*p*-phenylenediamine (TMPD), and ascorbate. No differences were noted in lung homogenate C-I function between any of the 4 groups of mice. In contrast, mice in the hyperoxia T3 group had increased C-IV respiration compared with mice in the hyperoxia vehicle group, although no differences were noted in the C-IV respiration of lung homogenates from normoxic mice given T3 or saline vehicle (Figure 3E).

To assess cell-specific effects of T3 supplementation on mitochondrial function, AT2s and NMLFs isolated from newborn mice were exposed to hyperoxia for 72 hours in cell cultures. Both basal and maximal OCR were higher in AT2s exposed to hyperoxia that were treated with T3 compared with untreated cells (Supplemental Figure 4, A and B). NMLFs treated with T3 and exposed to hyperoxia had higher basal OCR (but similar maximal OCR) when compared with untreated cells (Supplemental Figure 4C).

**Figure 4:**
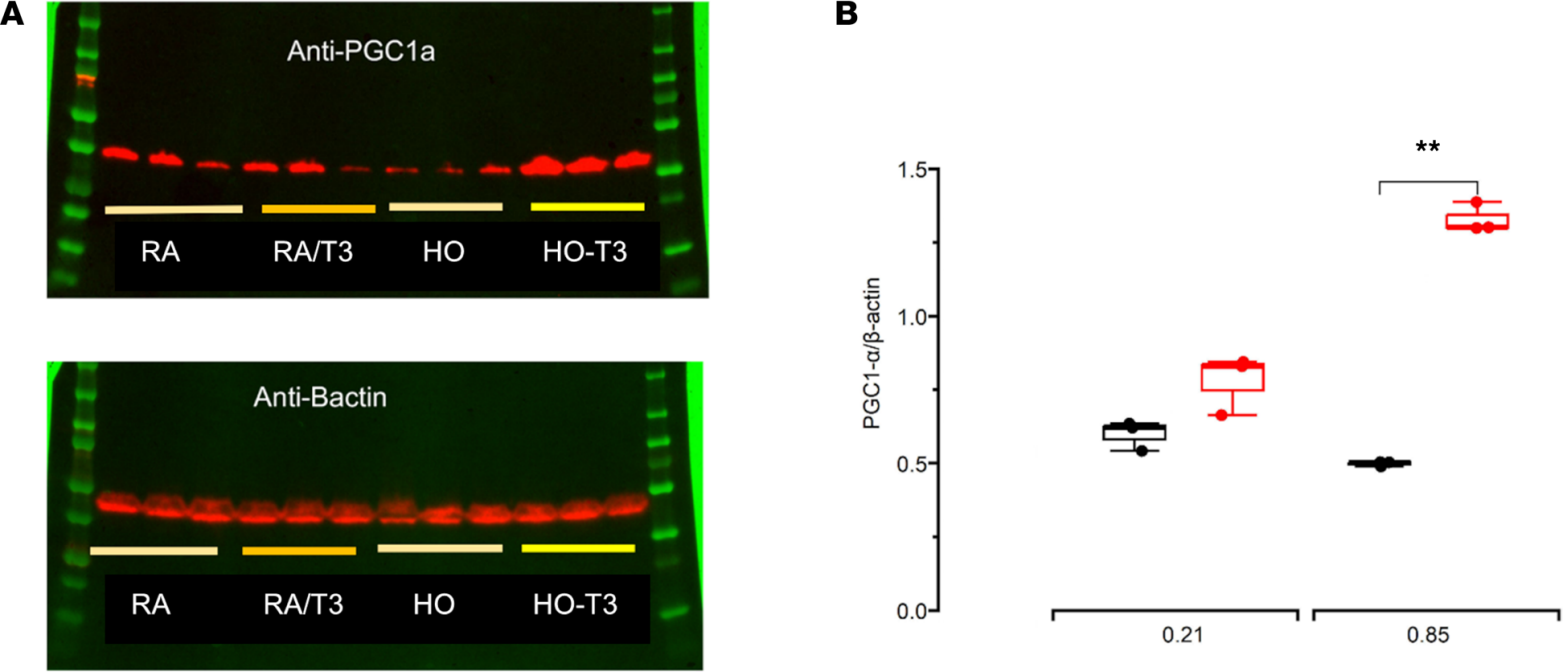
Lung PGC-1α measurements. (**A**) Western blots of PGC-1α protein content in lung homogenates from newborn mice exposed to normoxia (21% O_2_) or hyperoxia (85% O_2_) and vehicle or T3. (**B**) Densitometry measurements of PGC-1α content normalized to β-actin. *N* = 3 per group. All data were analyzed by 2-way ANOVA or Kruskal-Wallis tests, followed by post hoc analyses. Box represents median/IQR; whiskers represent maximum and minimum values. **P < 0.005. RA, room air (normoxia).

Peroxisome proliferator–activated receptor γ coactivator 1α (PGC-1α) is a master regulator of mitochondrial biogenesis, metabolic function, and cellular oxidative phosphorylation. Since THs are also capable of inducing mitochondrial biogenesis, we tested the hypothesis that the improved pulmonary bioenergetics noted in T3-exposed newborn mice was associated with increased PGC-1α expression. Lung homogenates from mice exposed to hyperoxia that were treated with T3 were noted to have increased PGC-1α compared with mice not supplemented with T3 (Figure 4). T3 also increased expression of some transcriptional upregulators of PGC-1α, such as myocyte-enhancing factor 2A (*Mef2a*) and forkhead box O1 (*Foxo1*), but not others, such as cAMP-responsive element-binding protein 1 (*Creb1*), in lung homogenates of mice exposed to hyperoxia (Supplemental Figure 5). Additionally, T3 was noted to increase PGC-1α expression in hyperoxia-exposed AT2s (Supplemental Figure 6, A, D, and G) but had no such effect in hyperoxia-exposed NMLFs (Supplemental Figure 7A), indicating that T3-induced changes in PGC-1α expression varied with cell type in lungs of mice exposed to hyperoxia.

### T3 decreases apoptosis and increases differentiation of AT2s exposed to hyperoxia.

To test whether T3 exposure changed apoptosis rates in AT2s, we conducted flow cytometry assays based on measurement of phosphatidylserine fluorescence. AT2s treated with T3 that were exposed to hyperoxia (as well as AT2s exposed to normoxia) had lower apoptosis rates when compared with untreated AT2s (Supplemental Figure 8, A and B). Cellular proliferation rates were higher in AT2s exposed to normoxia that were treated with T3 compared with untreated AT2s, but this difference was not noted in cells exposed to hyperoxia (Supplemental Figure 8C). No changes in apoptosis rates were noted in hyperoxia-exposed NMLFs that were treated with T3 compared with untreated cells (34 ± 5% vs. 30 ± 3%, *P* > 0.05).

T3 also increased expression of proteins such as podoplanin (PDPN or T1α) and aquaporin 5 (AQP5), which are considered to be specific markers of alveolar epithelial cells (AT1s), in AT2 cultures exposed to hyperoxia for 5 days, indicating that it could help maintain differentiation of AT2s to AT1s in newborn mice exposed to hyperoxia (Supplemental Figure 6, A–C, E, and F) (18).

### T3 increases OCR of UC-MSCs obtained from ELBW infants.

**Figure 5:**
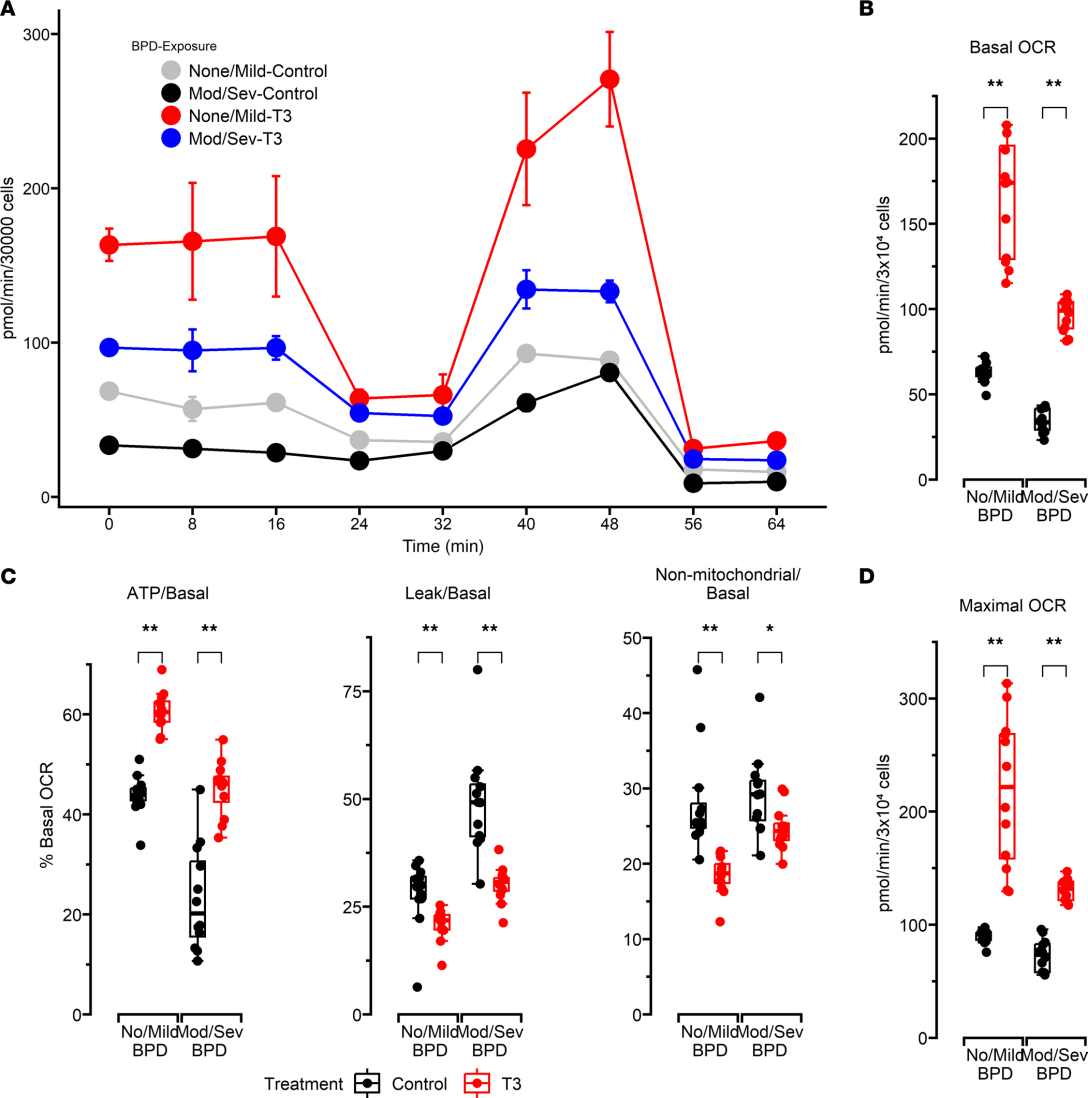
Bioenergetic measurements in MSCs exposed to vehicle or T3. (**A**) Representative plot of a typical extracellular flux assay conducted using a Seahorse XF96 flux analyzer. A total of 30,000 cells were seeded per well, and MSC oxygen consumption was measured in the presence of various mitochondrial effectors. All raw values are per 30,000 cells. (**B**) Basal OCR. (**C**) ATP-linked OCR, proton leak, and nonmitochondrial OCR presented as percentages of basal OCR. (**D**) Maximal OCR measured after FCCP introduction. *N* = 12 per group. All data were analyzed by 2-way ANOVA or Kruskal-Wallis tests, followed by post hoc analyses. Box represents median/IQR; whiskers represent maximum and minimum values. *P < 0.05; **P < 0.005.

MSCs isolated from umbilical cords of ELBW infants who died or developed moderate to severe BPD and infants who survived with no or mild BPD were exposed to vehicle (cell culture media) or T3 for 48 hours and then to mediators that inhibit various components of the mitochondrial ETC. Vehicle-exposed UC-MSCs from ELBW infants who died or developed moderate to severe BPD had lower basal OCR, ATP-linked fraction of basal OCR, and maximal OCR when compared with UC-MSCs from infants who survived with no or mild BPD. Nonmitochondrial OCR was similar between both groups. T3 exposure increased basal OCR, ATP-linked fraction of basal OCR, and maximal OCR of MSCs from both groups, indicating that T3 supplementation may improve MSC bioenergetics even in preterm infants without BPD whose lung development is ongoing (Figure 5). However, despite T3 supplementation, UC-MSCs from infants who died or developed moderate to severe BPD continued to have lower oxygen consumption versus UC-MSCs from those who survived with no or mild BPD (mean ± SEM; basal OCR: 96 ± 4 vs. 165 ± 12 pmol/min/3 × 10^4^ cells; maximal OCR: 131 ± 4 vs. 218 ± 25 pmol/min/3 × 10^4^ cells; and ATP-linked/basal OCR: 0.45 ± 0.02 vs. 0.60 ± 0.01; *P* < 0.005 for all comparisons, as noted in Supplemental Figure 9).

Notably, this increase in MSCs’ bioenergetic function caused by T3 supplementation was not accompanied by increased PGC-1α expression or mitochondrial content markers such as citrate synthase and TOM20. Similarly, although T3 also decreased proton leak in MSCs from infants who died or developed moderate to severe BPD as well as those who survived with no or mild BPD, there was no change in the expression of uncoupling protein 2 (*UCP2*), which is considered to be an important mediator of ETC uncoupling, indicating that other members of the UCP family such as *UCP1* or *UCP3* could play a role in causing these differences (Figure 5C and Supplemental Figures 9 and 10).

### T3 increases PGC-1α in murine lungs and mitochondrial membrane potential of NMLFs and MSCs exposed to hyperoxia.

**Figure 6:**
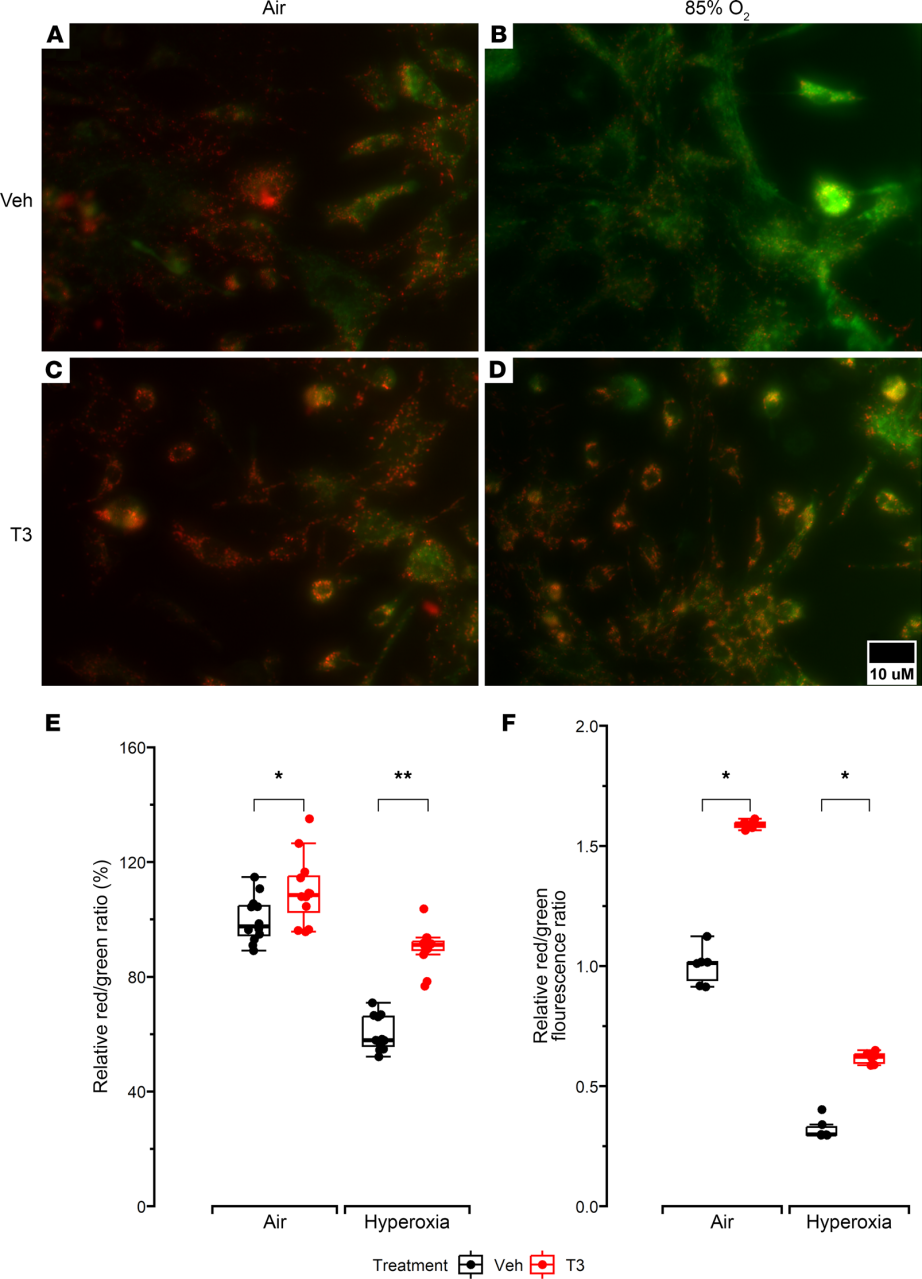
Measurements of ΔΨ_m_. (**A**–**D**) Representative photographs of JC-1–stained NMLFs that were previously treated with vehicle (cell culture media) or T3 and air or hyperoxia (85% O_2_). Dye accumulation in mitochondria was detected by fluorescence microscopy (aggregate red form with absorption/emission of 585/590 nm and green monomeric form with absorption/emission of 510/527 nm). (**E**) Box-and-whisker plot shows red/green fluorescence ratio of JC-1 dimers to monomers in NMLFs stained with JC-1. An increased ratio was considered to represent increased ΔΨ_m_. A minimum of 10 images were obtained for analysis from at least 6 mice per group. *N* = 3 per group. (**F**) Relative red/green fluorescence intensity ratio of JC-1–stained MSCs obtained from ELBW infants that were treated with vehicle (cell culture media) or T3 and air or hyperoxia (85% O_2_). *N* = 6 infants per group. All data were analyzed by 2-way ANOVA or Kruskal-Wallis tests, followed by post hoc analyses. Box represents median/IQR; whiskers represent maximum and minimum values. *P < 0.05; **P < 0.005.

Mitochondrial membrane potential (ΔΨ_m_) is a critical mediator of cellular ATP generation and bioenergetic efficiency. Hence, the effects of T3 on ΔΨ_m_ were measured both in NMLFs and in UC-MSCs from ELBW infants by measuring changes in the fluorescence intensity of the dye JC1. Lower numbers of NMLFs with red dimeric form of JC-1 (representing normal ΔΨ_m_) in contrast to cells with green JC-1 monomers (representing a depolarized mitochondrial membrane) were noted in the hyperoxia T3 group compared with the hyperoxia vehicle group, indicating that NMLFs exposed to T3 are able to better maintain mitochondrial polarity when exposed to hyperoxia than untreated NMLFs. An increased JC1 dimer/monomer ratio was also noted in the hyperoxia vehicle group compared with the air vehicle group, although this difference was less pronounced for this comparison (Figure 6, A–E). We also noted lower *UCP2* expression in NMLFs exposed to hyperoxia and treated with T3, indicating that this could be a possible mechanism for the higher ΔΨ_m_ noted in these cells compared with untreated NMLFs (Supplemental Figure 7, A–C).

T3 supplementation also increased relative red/green fluorescence (measured using spectrophotometry) in MSCs exposed to T3 versus MSCs exposed to vehicle (DMSO) under both normoxic and hyperoxic conditions (Figure 6F), indicating that T3 supplementation helps preserve ΔΨ_m_ in cells exposed to hyperoxia. However, as noted above and in contrast to its effects in NMLFs, T3 did not change *UCP2* expression in MSCs obtained from ELBW infants (Supplemental Figure 10).

### Neonatal hypothyroxinemia is associated with death or moderate to severe BPD.

NBS TT4 levels at 1–3, 7–10, and 28 days of age; gestational age (GA); BW; race; maternal hypertension; and Apgar scores at 5 and 10 minutes were collected from 195 ELBW infants admitted to UAB between April 2017 and January 2019 (discovery cohort). Of these, 148 infants (76%) survived with no or mild BPD, and 47 (24%) died or developed moderate to severe BPD. As expected, infants who died or developed moderate to severe BPD were more premature and weighed lower at birth compared with infants who survived with no to mild BPD. GA correlated weakly with NBS TT4 at 1–3, 7–10 and 28 days of age (0.30, 0.34, and 0.30, respectively; *P* < 0.0001 for all comparisons) whereas BW correlated moderately with NBS TT4 at these different ages (0.44, 0.45, and 0.37 respectively; *P* < 0.0001 for all comparisons) for the infants in this cohort.

**Figure 7:**
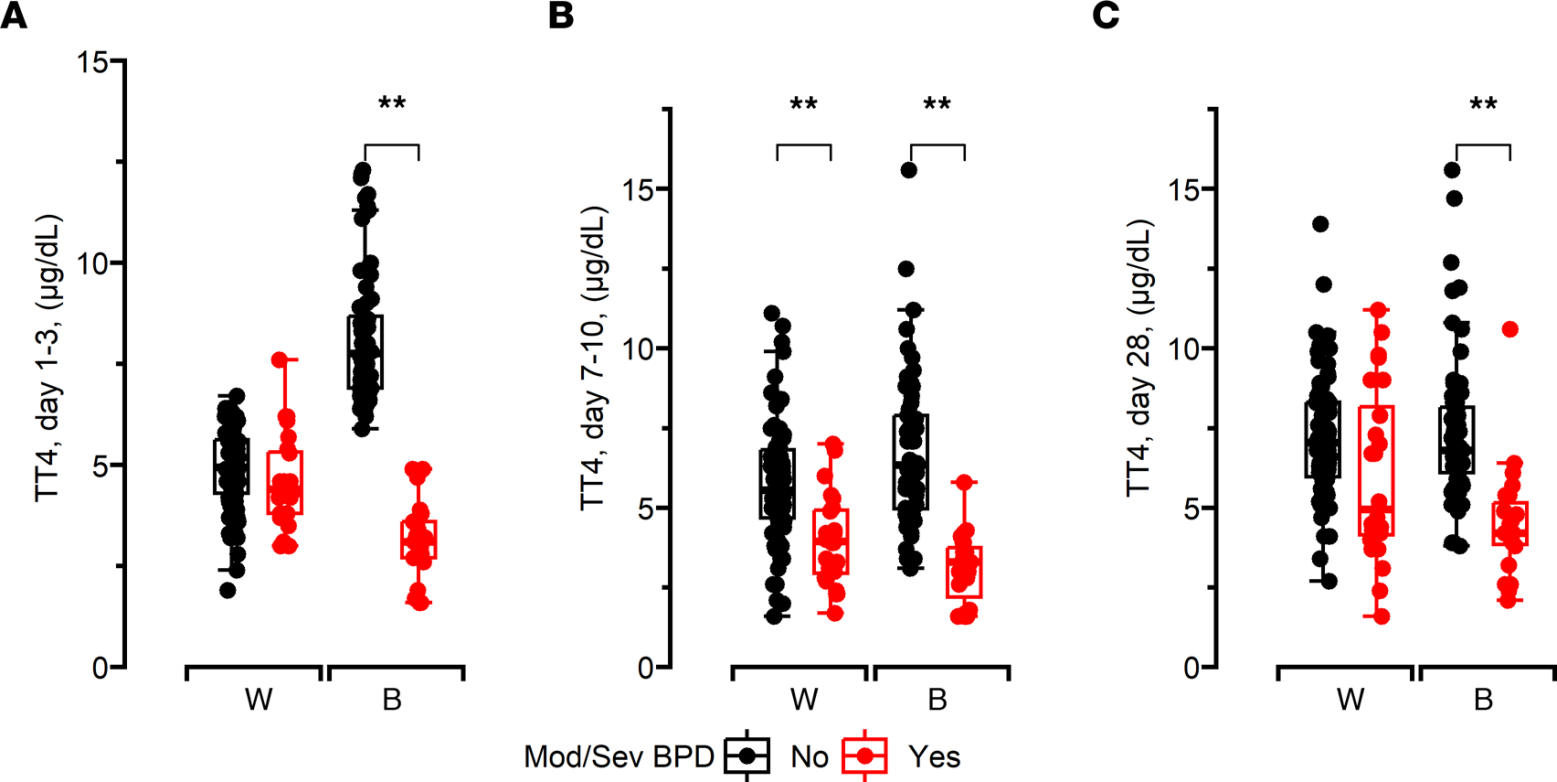
NBS TT4 measurements of 195 White and Black infants. Values obtained at 1–3 (**A**), 7–10 (**B**), and 28 days of life (**C**). All data were analyzed by 2-way ANOVA or Kruskal-Wallis tests, followed by post hoc analyses. Box represents median/IQR; whiskers represent maximum and minimum values. **P < 0.005. Mod/sev BPD, moderate to severe BPD or death before discharge; W, White; B, Black.

Bivariate analyses (Wilcoxon rank-sum and χ^2^ tests) showed that ELBW infants who died or developed moderate to severe BPD had significantly lower TT4 levels at 1–3, 7–10, and 28 days of age compared with infants who survived with no or mild BPD (Table 1). Race (based on documented maternal race) modified this association between NBS TT4 and BPD severity. Black infants (but not White infants) with moderate to severe BPD had lower NBS TT4 at 1–3 days of life and at 28 days when compared with those with no or mild BPD. At 7–10 days of life, NBS TT4 levels were lower in both Black and White infants with moderate to severe BPD (Figure 7).

**Figure 8:**
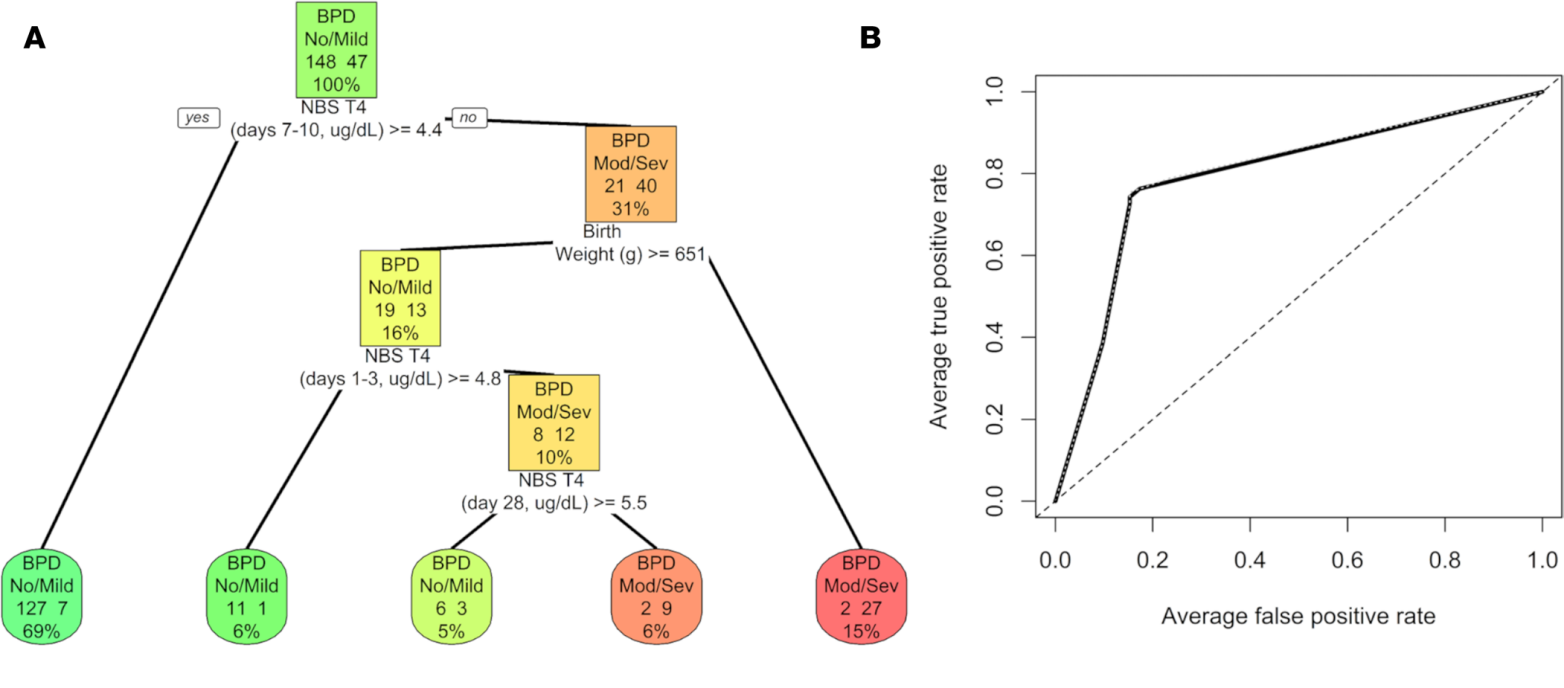
Classification tree to predict risk for moderate to severe BPD. (**A**) Classification tree constructed using data from discovery cohort of 195 infants to predict risk for moderate to severe BPD or death showed that NBS TT4 at 7–10 days of life and BW as the most predictive variables. (**B**) ROC obtained for the model when used to predict BPD severity in the validation cohort of 129 infants. ROC, receiver operating characteristics; mod/sev BPD, moderate to severe BPD or death before discharge.

In addition to these bivariate analyses, a classification and regression tree (CART) model (which included other variables such as GA, BW, race, maternal hypertension, and Apgar scores) was also constructed to determine cutoff points for NBS TT4 values that best predicted risk for death or moderate to severe BPD for infants in this cohort. The CART model identified an NBS TT4 less than 4.4 mg/dL at 7–10 days of life and BW less than 651 g as the most predictive variables of risk for moderate to severe BPD in this cohort. When used to predict BPD severity in another group of ELBW infants admitted to UAB between May 2020 to May 2021 (validation cohort, Table 2) for whom NBS TT4 values, GA, BW, and BPD status were available, the model had 84% sensitivity, 71% specificity, and an AUC of 79% (Figure 8). Infants with moderate to severe BPD who had lower MSC oxygen consumption also had lower TT4 values in the first 28 days of life when compared with infants with no or mild BPD and higher MSC oxygen consumption (Table 3).

## Discussion

Oxidative stress is a significant risk factor for BPD (19). Mitochondria are major mediators of cellular response to oxidative stress owing to their role as major sources of oxidants and because ETC components and mitochondrial DNA are highly susceptible targets of oxidative damage (20, 21). Inhibition of mitochondrial oxidative phosphorylation has been observed to produce BPD-like lung injury in animal models, and hyperoxia is known to induce mitochondrial dysfunction in AT2s derived from mice (6, 7). In previous studies, we found that lower umbilical venous endothelial cell basal and maximal OCR at birth are associated with increased BPD risk in ELBW infants and that newborn mitochondrial-nuclear exchange mice exposed to hyperoxia that developed severe lung injury also had decreased NMLF (resident fibroblast) oxygen consumption (8, 9). Recent studies indicate that aerosolized T3 decreases bleomycin-induced fibrosis in adult mice by improving mitochondrial function in AT2s and through alterations in mitophagy (16, 17). The current study extends these observations further by finding that nonsystemic delivery of TH decreases alveolar/airway injury and improves isolated ETC complex, mitochondrial, alveolar-epithelial, and fibroblast-mitochondrial function in a hyperoxia-induced BPD model in newborn mice. The increased expression of PGC-1α and its transcriptional upregulators such as *Mef2a* and *Foxo1* in lung homogenates (and PGC-1α in AT2s) from hyperoxia-exposed T3-treated mice compared with hyperoxia-exposed saline-treated mice suggests that lung injury caused by oxidative stress in the developing lung could be mitigated through a TH-induced increase in pulmonary mitochondrial biogenesis (22, 23). Our finding that T3 supplementation helps to improve AT2 differentiation to AT1s during hyperoxia exposure indicates that a T3-induced increase in cellular bioenergetic function and reduction in apoptosis could contribute to improved repair of lung injury during hyperoxia.

MSCs and fibroblasts are critical drivers of lung development (24). Mitochondrial transfer to alveolar epithelial cells (AECs) and bronchial epithelial cells has been proposed as a rescue mechanism in acute lung injury and may also be relevant to the pathogenesis of respiratory illnesses such as chronic obstructive pulmonary disease and asthma (17, 25). Since their proliferation, self-renewal, and pluripotency are significantly impaired by oxidative stress, MSC mitochondrial dysfunction may play a critical role in BPD pathogenesis (26–28). MSCs obtained from umbilical cords have also shown therapeutic potential in BPD, which makes them a biologically relevant choice to study the effects of TH on cellular mitochondrial function in ELBW infants (24, 29–31). This study found that infants with moderate to severe BPD who had lower NBS TT4 levels also had decreased MSC mitochondrial oxygen consumption. Therefore, our finding that T3 improved MSC mitochondrial bioenergetics suggests that TH supplementation–induced improvements in mitochondrial function and transfer of healthier mitochondria from resident and nonresident MSCs to other lung cells such as AECs might be a mechanism responsible for the improvement in lung histology and mechanics noted in the mice supplemented with i.n. T3. Our findings that differences in bioenergetic function induced by T3 in MSCs were not associated with changes in PGC-1α expression or cellular mitochondrial content indicate that mechanisms other than increased mitochondrial biogenesis, such as changes in mitochondrial efficiency caused by differential ETC component function, could be the underlying mechanism for T3-induced changes in MSC mitochondrial function. T3-induced improvement in NMLF mitochondrial function may also be associated with changes in ROS generation and downstream signaling effects on profibrotic pathways that could reduce deposition of extracellular matrix around the alveoli leading to improved compliance, in hyperoxic as well as normoxic mice, as has been previously noted in idiopathic pulmonary fibrosis and newborn mice supplemented with vitamin D (32, 33).

Increased mitochondrial oxidative stress and induction of autophagy is known to decrease the activity of membrane channels such as Na^+^, K^+^-ATPase, and epithelial Na^+^ channels that participate in alveolar fluid resorption and are major mediators of early onset respiratory distress in preterm infants (34, 35). T3 supplementation increases alveolar fluid clearance in hyperoxia-exposed lungs, indicating that improved mitochondrial health due to T3 activity could be an underlying mechanism for the decreased respiratory distress noted in small, randomized trials and cohort studies that have found that hypothyroidism may mediate respiratory outcomes in moderately preterm infants (36–39). Since pulmonary surfactant production in lamellar bodies of AECs can be modified by mitochondrial functional integrity, improved mitochondrial function through direct i.n. T3 supplementation may be beneficial not only in extremely premature infants at risk for BPD but also in moderately preterm infants who are at risk for milder lung disease such as respiratory distress syndrome (40, 41). Finally, downregulation of DIO2 — an iodothyronine deiodinase that converts T4 to T3 that has recently been identified as a regulator of mitochondrial function — has been noted to increase pulmonary fibrosis in mice (16, 42). Since excessive fibrosis contributes significantly to BPD pathogenesis, studies of pulmonary DIO2 gene expression and activity in newborn mice and ELBW infants could provide more clarity regarding the role of TH-induced mitochondrial functional changes in hyperoxia-induced neonatal lung injury.

Neonatal hypothyroxinemia within the first week of life is known to be associated with respiratory morbidities in moderately preterm infants, and recent studies in ELBW infants from South Korea and Turkey have shown that it is also associated with increased risk for BPD (43, 44). However, clinical studies of systemic TH supplementation in preterm infants have provided conflicting results, and a systematic review has concluded that further research is required (45, 46). This current study conducted in a US cohort of ELBW infants also supports such findings by showing that neonatal hypothyroxinemia over the first month of life is associated with increased BPD risk in these infants using bivariate analyses as well as a classification tree model that identified NBS TT4 levels at 7–10 days of life as a strong predictor of BPD risk. Our finding that variations in NBS TT4 levels associated with BPD risk are more significant in Black infants than in White infants (Table 1) also implies that TH supplementation (whether systemic or inhaled) may benefit certain subsets of infants more than others. Finally, GA and BW correlated only moderately with NBS TT4 levels in this study, indicating that serum TH levels may remain relatively stable at 23–28 weeks of GA, a finding that has also been noted in another study of blood spot T4 levels in newborn infants (47).

Our study has a few limitations. First, we utilized readily available NBS TT4 values to assess hypothyroxinemia in ELBW infants but did not evaluate thyroid function in a prospective cohort of newborn infants. However, NBS TT4 values are readily available and do not require any additional testing. We also avoided including infants with only mild BPD per the NIH *Eunice Kennedy Shriver* National Institute of Child Health and Human Development (NICHD) definition in our high-risk group. Therefore, our finding that transient hypothyroxinemia during the first 28 days of life was a marker for moderate to severe BPD risk adds to existing evidence that supports the need for a large multicenter randomized trial to determine the effectiveness of pulmonary and/or systemic TH supplementation in reducing BPD risk in ELBW infants. Next, while we were able to measure mitochondrial function in AT2s and NMLFs as well as in whole lung homogenate–isolated mitochondria and AT2 differentiation in cell cultures exposed to T3, assessment of bioenergetic function in other lung cell populations, such as vascular endothelial and bronchial epithelial cells, as well as comparative imaging studies of freshly cultured AECs versus cells treated with T3 over time, would help to provide a more complete picture of T3-induced changes in pulmonary bioenergetics and AEC differentiation in hyperoxia-induced neonatal lung injury models. Additionally, we did not measure changes in serum TH levels secondary to i.n. T3 delivery, but only minimal systemic deposition secondary to swallowing during the instillation was observed in these mice. Similarly, while safety of aerosolized T3 for acute respiratory distress syndrome is currently being evaluated in a preclinical study, in this study we ensured that all mice received the same dose of T3 (0.1 mcg/g weight) by directly instilling it i.n., which was found to be well tolerated without signs of systemic toxicity (48). Reduced expression of UCP2 could be the underlying mechanism for the preserved ΔΨ_m_ noted in hyperoxia-exposed NMLFs, but there was no change in UCP2 expression in MSC, which were also noted to have relatively well-preserved ΔΨ_m_ during hyperoxia, indicating a need to explore changes in expression of other members of the uncoupling protein family such as UCP1 and UCP3 (49). Finally, it is well known that preterm birth is often accompanied by widespread and generalized alterations of the hypothalamic/pituitary axis, and we hope to study the impact of such changes on other endocrine effectors such as growth hormone and IGF and their impact on lung growth and development in subsequent studies (50).

In summary, this study has found that TH supplementation protects against lung injury and pulmonary mitochondrial dysfunction in newborn mice given i.n. T3 while exposed to hyperoxia and also improves mitochondrial function in NMLFs and MSCs from infants. We also found that neonatal hypothyroxinemia is associated with BPD severity in ELBW infants. Further studies to assess the effects of T3 on mitochondrial biogenesis, differentiation, and apoptosis in lung cells such as AT2s using imaging, proteomic, and genomic techniques as well as through animal models such as DIO2-KO mice and clinical trials of nebulized T3 could enhance our understanding of the mechanisms and impact of TH function in lungs and airways in early life.

## Methods

### Murine BPD model and T3 supplementation.

Newborn C57BL/6 mice obtained from Jackson Laboratory that were exposed to air or hyperoxia (85% oxygen) were administered a daily i.n. instillation of vehicle (saline) or 0.1 μg/g of T3 (L-3,3′,5-triiodothyronine, Sigma-Aldrich) between P3 and P14. Following these exposures, mice were anesthetized for lung function assessments and euthanized for lung injury and mitochondrial studies.

### Murine lung injury assessments.

Pulmonary mechanics (compliance and resistance) were assessed using a flexiVent (SciReq). Lung structure was assessed by measuring the MLI and RAC in H&E-stained sections as previously described (51, 52).

### Murine lung mitochondrial isolation.

Lung mitochondria were isolated following a modification of the method of Rasmussen et al. using a custom homogenizer (53). A freshly isolated lung sample was weighed, carefully cut into 1 mm cubes with a 9 mm razor blade by hand after immediately being put into 6 mL of ice-cold B1 with EGTA isolation buffer containing protease inhibitor. The samples were homogenized with a Craftsman drill press at 55*g* using a customized Wheaton mortar and pestle, designed to standardize optimal and consistent yields and functionality in mitochondrial preparations (54). The samples were maintained at 0°C–1°C in a custom-built clear water-jacketed chamber. Homogenization included 8 slow passes of the pestle at 30 seconds for each down stroke, 30 seconds stopping of rotation once to the bottom, and 30 seconds on the upward pass. Homogenates were centrifuged at 600*g* for 10 minutes at 4°C. The supernatants were transferred to another ice-cold centrifuge tube and centrifuged at 10,000*g* for 10 minutes at 4°C. The pellet was then rinsed with 50 μL of ice-cold isolation buffer, and the supernatant removed. The final pellet was gently resuspended in 100 μL of isolation buffer and protein concentration determined by Lowry quantification.

### Murine lung mitochondrial bioenergetics.

Mitochondrial respiration assays, using 300 μg of freshly isolated mitochondria per sample, were performed using high-resolution respirometry by measuring oxygen consumption in a 2-channel respirometer (Oroboros Oxygraph-2k with DatLab software). Seventy percent ethanol was run in both chambers for a minimum of 30 minutes, and the chambers were calibrated after a stable air-saturated signal was obtained before every experiment. Reactions were conducted at 37°C in a 2 mL chamber containing air-saturated mitochondrial respiration buffer (Oroboros Instruments) under continuous stirring with 300 μg of mitochondrial protein present in the assay. Respiration rates were measured using various substrates, uncouplers, and inhibitors of oxidative phosphorylation. Determination of state 2 or the leak respiration rate was made in the presence of excess substrates glutamate (15 mM) and malate (5 mM). State 3 was measured after the addition of ADP (2.5 mM) and after the addition of succinate (10 mM). Oligomycin (3.2 μM) was added to measure state 4 respiration. Uncoupled respiration was measured using increasing doses of FCCP from 1 to 3 μM. Respiration after the inhibition of C-I was measured by adding rotenone (0.5 μM), inhibition of C-II by adding malonate (5 mM), and inhibition of C-III by adding antimycin-A (5 μM). Additionally, cytochrome *c* was added to assess outer membrane integrity and TMPD (0.5 mM) and ascorbate (2 mM) to measure maximal C-IV activity, which also approximates mitochondrial content. A typical experiment is shown in Figure 3.

### AT2, NMLF, and UC-MSC isolation.

To isolate AT2s, newborn mouse lungs were injected with dispase and agarose gel and incubated in dishes with dispase for 30 minutes. Next, dispase-treated lungs were immersed in 5 mL of DMEM with DNase and 1 μg/mL of anti-CD16/32 Ab (Thermo Fisher Scientific, catalog 14-0161-82) and disintegrated and filtered through 30–100 μM nylon meshes. The filtrate was then centrifuged for 10 minutes at 160*g* at 4°C. After treatment with erythrocyte lysis buffer (Abcam, catalog ab204733) and subsequent centrifugation, the resulting cell pellet was incubated in 2 mL of a primary Ab cocktail made up of anti-F4/80, anti-CD11b, anti-CD11c, and anti-CD45 (Abcam, catalog ab6640, ab8878, ab33483, and ab10558, respectively) in DMEM for 10 minutes; washed; filtered through a 50 μM mesh, and sorted using a BD Biosciences FACSMelody cell sorter using a gating strategy that selected for cells negative for the primary Abs. The resultant AT2 cell pellet was suspended and incubated in airway epithelial media (Promocell) for up to 7 days and/or used for experiments as needed (55).

To isolate NMLFs, mice lung explants were suspended in PBS with 0.5% trypsin and incubated with gentle stirring, and the dispersed cells were separated from debris by filtration through a sterile gauze. The resulting cell suspension was then washed with PBS and resuspended in DMEM with 10% FBS and incubated for 48 hours at 37°C to obtain a monolayer of NMLFs, which was then passaged or used for experiments as needed (56).

UC-MSCs were isolated by following a protocol we have described previously using umbilical cord segments that were collected from infants born at or before 28 weeks’ gestation after informed consent was obtained from their mothers (21).

### Intact cell bioenergetics assays.

MSCs, NMLFs, and AT2s were treated with vehicle (cell culture media) or T3 (0.8 ng/mL) for 48 hours. Then, 96-well plates were seeded with 30,000 MSCs/well, and the OCRs were measured using an XF analyzer (Agilent Technologies) using various mitochondrial effectors as previously described. (8) Briefly, basal OCR was measured first using glucose present in assay media as the substrate. Oligomycin, which inhibits ATP generation at C-V of the ETC, antimycin-A, and rotenone, which block substrates from transferring electrons to the ETC at C-III and C-I, respectively, were added to measure ATP-linked OCR, proton leak (oxidation uncoupled from phosphorylation), and nonmitochondrial OCR (typically considered to be from enzymatic oxidant generation) — all of which constitute basal OCR. MSC maximal OCR was measured using FCCP, a protonophore that maximizes proton leak (Figure 4A).

### NMLF ΔΨ_m_ measurement.

NMLFs were seeded in 24-well plates with 5 × 10^4^ cells per well in standard growing culture media. The cells were allowed to attach overnight (18–24 hours). The fibroblasts were exposed to air (21%) or hyperoxia (85%) while being treated with either vehicle (standard culture media) or T3 (250 μL/well of 0.8 ng/mL T3 solution) for 48 hours. JC-1 staining was performed by adding 250 μL of 20 μM JC-1 (Mitochondrial Membrane Potential Assay Kit, Abcam, catalog ab113850) solution to each well. The plates were incubated for 10 minutes at 37°C in the dark. After incubation, the plates were washed twice with 250 μL/well of 1× Dilution Buffer Solution from the kit. The samples were immediately analyzed with a fluorescence microscope with a “dual-bandpass” filter (Nikon TE2000-U, Nuance Multispectral Imaging Camera System CRI Model N-MSI-FX). The images taken by microscopy were evaluated to quantify the proportion of red fluorescence (aggregate red form with absorption/emission of 585/590 nm) to green fluorescence (green monomeric form with absorption/emission of 510/527 nm). The red/green fluorescence ratio was used to assess ΔΨ_m_, and decreased red to green fluorescence was considered to be indicative of mitochondrial depolarization.

To assess MSC ΔΨ_m_, cells were seeded at 1 × 10^4^ cells on a dark 96-well plate and allowed to adhere for 24 hours. The following day, cells were washed once with PBS and incubated with JC-1 dye (2 μM) at 37°C for 10 minutes. Cells were then washed twice with PBS, and red/green fluorescence intensity ratio was measured using a fluorescence spectrophotometer (SpectraMax i3X) at excitation 475 nm and emission 530/590 nm to determine ΔΨ_m_.

### Mouse serum T4 estimation.

A T4 ELISA kit (Sigma-Aldrich, catalog SE120090) was used to create standard curves and to measure serum T4 levels in frozen serum samples separated from 2 mL of blood collected from mice used in the study following manufacturer’s instructions (57).

### Apoptosis and proliferation assays in AT2s.

To measure apoptosis, AT2s isolated from newborn mouse lungs were exposed to normoxia or hyperoxia and treated with saline or T3 (0.8 ng/mL) for 72 hours and then stained with Apopxin green and 7-aminoactinomycin D (7-AAD) using an Apoptosis/Necrosis Detection Kit (Abcam, catalog ab176749). An LSRII flow cytometer (BD Biosciences) was used to quantify Apopxin green (Ex/Em = 490/525 nm) and 7-AAD (Ex/Em = 550/650 nm) fluorescence, and FlowJo software 10.8 (BD Biosciences) was used to analyze the results as described by the manufacturer (58).

AT2 proliferation was measured in cells exposed to normoxia or hyperoxia and treated with saline or T3 (0.8 ng/mL) for 72 hours using a CyQuant cell proliferation assay kit (Invitrogen, catalog MP 07026) and a fluorescence spectrophotometer (SpectraMax i3X) according to manufacturer’s instructions (59).

### Western blots for PGC-1α, UCP2, CS, TOM20, and AEC markers.

Mouse lung tissue was weighted and cut to get an approximately 20 mg piece, which was then placed in 250 μL of cold RIPA buffer (Alfa Aesar, catalog J62885) and homogenized on ice with a glass tissue grinder. After the freeze-thaw step, the lysate was clarified by centrifuging at 6,000*g* for 10 minutes at 4°C, and the supernatant was mixed with 4× Laemmli sample buffer for Western blotting according to standard procedures. A total of 30–60 μg of total protein of the samples was separated on 4%–20% Criterion TGX Gels (Bio-Rad, catalog 5678093), transferred to PVDF membranes (Trans-Blot Turbo System, Bio-Rad), and probed with Abs against PGC-1α (Novus Biologicals, catalog NBP1-04676) with the dilution 1:1,000 and β-actin (Cell Signaling Technology, catalog 5125S) as loading control. Membranes were developed using Clarity ECL Substrate (Bio-Rad, catalog 1705060) and visualized with ChemiDoc imaging system (Bio-Rad). Densitometry analysis of band intensities was performed using Image Lab software.

AT2s, NMLFs, and MSCs were lysed in RIPA buffer (Alfa Aesar, catalog J62885), the lysate was centrifuged at 6,000*g* for 10 minutes at 4°C, and then the supernatant was mixed with 4× Laemmli sample buffer for Western blotting according to standard procedures. Approximately 30–60 μg of total protein of the samples were separated on 4%–20% Criterion TGX Gels (Bio-Rad, catalog 5678093), transferred to PVDF membranes using Trans-Blot Turbo System (Bio-Rad), and probed with Abs against AQP5 (Invitrogen, catalog PA536529), PDPN (Invitrogen, catalog MA535784), UCP2 (Cell Signaling Technology, catalog 50-204-9176), PGC-1α (Novus Biologicals, catalog NBP1-04676), TOM20 (Santa Cruz Biotechnology, catalog sc-17664), and β-actin (Cell Signaling Technology, catalog 5125S) as loading control. Membranes were developed using Clarity ECL Substrate and visualized with ChemiDoc imaging System. Densitometry analysis of band intensities was performed using Fiji 2.9 software. All real-time PCRs (qPCRs) were carried out using 3 independent samples per group.

### mRNA analysis for PGC-1α, PGC-1α pathway regulators, UCP2, and AEC markers.

mRNA expression was carried out if the corresponding Western blots showed significant differences. RNA was isolated from lung homogenates or AT2s and NMLFs with RNeasy Plus Mini Kit (QIAGEN, catalog 74136). The concentration and purity of the RNA were checked using SpectraMax i3x (Molecular Devices). Then, a total of 1 μg of RNA was reverse-transcribed to cDNA using iScript Reverse Transcription Supermix (Bio-Rad, catalog 1708840) following the manufacturer’s instructions, and cDNA was used as a template in the subsequent qPCR, which was performed using TaqMan Fast Advanced Master Mix (Thermo Fisher Scientific, catalog 4444556) with TaqMan gene expression assays for *PDPN* (Hs00366766_m1), *Aqp5* (Hs00387048_m1), *UCP2* (Hs01075227_m1), and PGC-1α (Hs00173304_m1) in AT2- and NMLF-derived mRNA and for *Mef2a* (Mm01319888_m1), *Foxo1* (Mm00490671_m1), and *Creb1* (Mm00501607_m1) in lung homogenate–derived mRNA according to the manufacturer’s instruction of Taqman Fast Advanced Master Mix on the Bio-Rad CFX96 system. To correct for differences in the amount of cDNA loading into qPCR reaction wells, Eukaryotic 18S rRNA Endogenous Control (Thermo Fisher Scientific, catalog 4310893E) was used to normalize the expression levels of the target genes. qPCR data were analyzed by applying the comparative Ct method (ΔΔCt). The results were presented as the fold-change in mRNA expression for targeted genes relative to controls. All qPCRs were carried out using 3 independent samples per group.

### Neonatal TT4 in infants.

TT4 from Alabama state NBS at P1–3, P7–10, and P28; BPD status (as defined by the NICHD consensus statement on BPD); GA; and BW were collected from the electronic health records of ELBW infants born at or before 28 weeks’ GA admitted to the UAB Regional Neonatal Intensive Care Unit between April 2017 and January 2019 and between May 2020 to May 2021. Infants with major congenital anomalies or unavailable NBS results were excluded. TT4 values were compared between infants who died or developed moderate to severe BPD and those who survived with no or mild BPD.

### Statistics.

Results are expressed as the mean ± SD or median (IQR). A 2-way ANOVA followed by 2-tailed Student’s *t* test (parametric) and Kruskal-Wallis test followed by Dunn’s test (nonparametric) were used to test for significant differences between individual pairwise comparisons. The χ^2^ test or Fisher’s exact test were used for categorical variables. Pearson’s and Spearman’s rank tests were used to test for correlations between continuous variables. *P* < 0.05 was considered significant and Bonferroni’s method was used for multiple-comparison correction. A stepwise logistic regression model was used to test the strength of association between BPD risk and risk factors. All analyses including were carried out using R 4.1.1.

### Study approval.

All protocols were approved by the IACUC and the IRB of the UAB and were consistent with the Public Health Service Policy on Humane Care and Use of Laboratory Animals (NIH Office of Laboratory Animal Welfare, 2002).

## Author contributions

BMV, TN, NK, JK, and NA conceived and designed the study. BMV, TN, SH, and RL performed the experiments. BMV, TN, NA, and JK analyzed the data. BMV, TN, SM, NA, and JK interpreted the results of the experiments. BMV, TN, NA, and JK prepared the figures. BMV, TN, NA, and JK drafted the manuscript. BMV, TN, SH, RL, SM, NK, NA, and JK revised and approved the final version of the manuscript.

## Supplementary Material

Supplemental data

## Figures and Tables

**Table 3:**
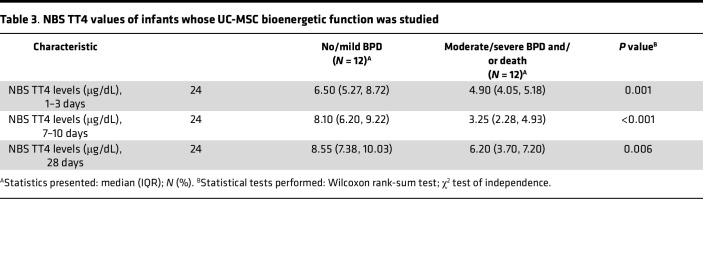
NBS TT4 values of infants whose UC-MSC bioenergetic function was studied

**Table 2:**
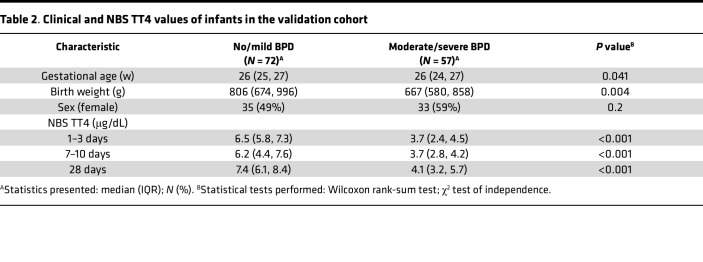
Clinical and NBS TT4 values of infants in the validation cohort

**Table 1:**
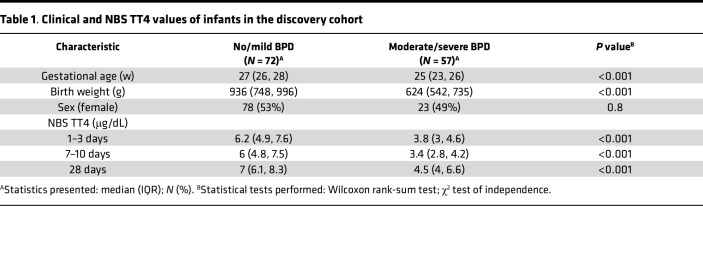
Clinical and NBS TT4 values of infants in the discovery cohort

## Version 1 03/14/2023

In-Press Preview

## Version 2 04/24/2023

Electronic publication
