# Supplementary material for: Thyroid hormone modulates hyperoxic neonatal lung injury and mitochondrial function
Source: JCI Insight. 2023 Apr 24;8(8):e160697. doi: 10.1172/jci.insight.160697 (PMC10243814; doi:10.1172/jci.insight.160697)

ONLINE DATA SUPPLEMENT

This appendix has been included to provide readers with additional information about the manuscript **Thyroid hormone modulates hyperoxic neonatal lung injury and mitochondrial function.**

Authors: Bianca M. Vamesu, Teodora Nicola, Rui Li, Snehashis Hazra, Sadis Matalon, Naftali Kaminski, Namasivayam Ambalavanan, Jegen Kandasamy

SUPPLEMENTAL FIGURE LEGENDS

Supplemental Figure 1: Serum T4 levels in newborn mice exposed to normoxia or hyperoxia. N = 6 mice per group. All data were analyzed by 2-way ANOVA or Kruskal-Wallis tests, followed by post hoc analyses. Box represents median/interquartile range, whiskers represent maximum and minimum values. * and ** represent p-value < 0.05 and < 0.005 respectively. T4 – Thyroxine

Supplemental Figure 2: Pressure-volume (PV) loops of lungs from mice obtained using Flexivent™. Dynamic lung compliance was noted to be highest in mice exposed to air & T3 and lowest in mice exposed to hyperoxia & saline vehicle.

Supplemental Figure 3: Additional comparisons for bioenergetic function and ETC complex activities of lung mitochondria shown in Figure 3. **A**: Representative Oxygraph experiment showing oxygen consumption rates measured in the presence of various substrates, uncouplers and inhibitors of oxidative phosphorylation: excess substrates glutamate and malate (state 2), addition of ADP and succinate (state 3), and Oligomycin (state 4). Uncoupled respiration was measured using increasing doses of 2-[2-[4-(trifluoromethoxy)phenyl] hydrazinylidene]-propanedinitrile (FCCP). Respiration after the inhibition of complex I was measured by adding rotenone, inhibition of complex II by adding malonate and inhibition of complex III by adding Antimycin A. Cytochrome c was added to assess outer membrane integrity and N, N,N',N',-tetramethyl-p-phenylenediaminepalmitylcarnitine TMPD and ascorbate to measure maximal complex

IV activity. **B**. Panel showing additional comparisons between mice exposed to saline or T3 and normoxia or hyperoxia. N = minimum of 6 mice/group. All data were analyzed by 2-way ANOVA or Kruskal-Wallis tests, followed by post hoc analyses. Box represents median/interquartile range, whiskers represent maximum and minimum values. * and ** represent p-value < 0.05 and < 0.005 respectively. NS – normal saline, T3 – triiodothyronine, OCR - oxygen consumption rate.

Supplemental Figure 4: Bioenergetic measurements in AT2 and NMLF exposed to vehicle or T3 and air or hyperoxia for 48 hours. 30,000 cells were seeded per well and MSC oxygen consumption was measured in the presence of various mitochondrial effectors. All raw values are per 30,000 cells. **A**: Basal OCR in AT2. **B**: Maximal OCR in AT2. **C**: Basal OCR in NMLF (T3 did not induce significant differences in NMLF maximal OCR). N = minimum of 3 mice per group. All data were analyzed by 2-way ANOVA or Kruskal-Wallis tests, followed by post hoc analyses. Box represents median/interquartile range, whiskers represent maximum and minimum values. * represents p-value < 0.05. Veh – vehicle, T3 – triiodothyronine, AT2 – type 2 alveolar epithelial cell, NMLF – neonatal murine lung fibroblast, OCR - oxygen consumption rate.

Supplemental Figure 5: qPCR analysis for PGC1-α regulators in mice lungs. **A**: myocyte enhancing factor 2A (*Mef2a*), **B**: forkhead box O1 (*Foxo1*) and **C**: cAMP responsive element binding protein 1 (*Creb1*) mRNA in lung homogenates of newborn mice exposed to normoxia (21% O_2) or hyperoxia (85% O_2) and vehicle or T3. N = 3 mice per group. All

data were analyzed by 2-way ANOVA or Kruskal-Wallis tests, followed by post hoc analyses. Box represents median/interquartile range, whiskers represent maximum and minimum values. * and ** represent p-value < 0.05 and < 0.005 respectively. Veh – vehicle, T3 – triiodothyronine.

Supplemental Figure 6: Mitochondrial and AT1 marker analysis in alveolar epithelial cells. AT2 cells from newborn mice were exposed to normoxia (21% O_2) or hyperoxia (85% O_2) and vehicle or T3. **A**: Western blots of podoplanin, aquaporin5 and PGC1-α content. **B-D**: Relative densitometry measurements of podoplanin, aquaporin5 and PGC1-α content normalized to β-actin. **E-G**: Relative expression of podoplanin, aquaporin5 and PGC1α mRNA. N = 3 mice per group. All data were analyzed by 2-way ANOVA or Kruskal-Wallis tests, followed by post hoc analyses. Box represents median/interquartile range, whiskers represent maximum and minimum values. * and ** represent p-value < 0.05 and < 0.005 respectively. AT1 –type 1 alveolar epithelial cells, AT2 – type 2 alveolar epithelial cells, Veh – vehicle, T3 – triiodothyronine.

Supplemental Figure 7: PGC-α and UCP2 marker analysis in NMLF. NMLF obtained from newborn mice were exposed to normoxia (21% O_2) or hyperoxia (85% O_2) and vehicle or T3. **A**: Western blots of UCP2 and PGC1-α content. **B**: Relative densitometry measurement of UCP2 content normalized to β-actin. **C**: Relative expression of UCP2 mRNA. N = 3 mice per group. All data were analyzed by 2-way ANOVA or Kruskal-Wallis tests, followed by post hoc analyses. Box represents median/interquartile range, whiskers

represent maximum and minimum values. * and ** represent p-value < 0.05 and < 0.005 respectively. NMLF – neonatal murine lung fibroblasts, Veh – vehicle, T3 – triiodothyronine.

Supplemental Figure 8: Apoptosis and proliferation assays in AT2 cells from mice. Newborn mice-derived AT2 cells were exposed to normoxia (21% O_2) or hyperoxia (85% O_2) and vehicle or T3 in culture. **A**: Representative flow cytometry experiment with Q3 showing fraction of cells positive for Apopxin green indicative of cells undergoing apoptosis. **B**: Apopxin+, 7-AAD- cell fraction and **C**: Proliferation assays in AT2 cells. N = 3 mice per group. All data were analyzed by 2-way ANOVA or Kruskal-Wallis tests, followed by post hoc analyses. Box represents median/interquartile range, whiskers represent maximum and minimum values. * and ** represent p-value < 0.05 and < 0.005 respectively. AT2 – type 2 alveolar epithelial cells, NMLF – neonatal murine lung fibroblasts, Veh – vehicle, T3 – triiodothyronine.

Supplemental Figure 9: Additional comparisons for the bioenergetic measurements in MSC exposed to vehicle or T3 shown in Figure 5. N = 12 per group. All data were analyzed by 2-way ANOVA or Kruskal-Wallis tests, followed by post hoc analyses. Box represents median/interquartile range, whiskers represent maximum and minimum values. * and ** represent p-value < 0.05 and < 0.005 respectively. T3 – triiodothyronine, MSC - mesenchymal stem cell, BPD - bronchopulmonary dysplasia, OCR - oxygen consumption rate.

Supplemental Figure 10: Mitochondrial protein expression in MSCs. **A**: Western blots of uncoupling protein 2 (UCP2), PGC1α, Citrate synthase (CS) and Translocase outer membrane 20 (TOM20) in MSCs from infants that were exposed to vehicle or T3. Densitometric analyses of **B:** UCP2, **C:** PGC1-α, **D:** CS and **E**: TOM20. N = 3 infants per group. All data were analyzed by 2-way ANOVA or Kruskal-Wallis tests, followed by post hoc analyses. Box represents median/interquartile range, whiskers represent maximum and minimum values. NS – not significant, Veh – vehicle, T3 – triiodothyronine, MSC – mesenchymal stem cell, NS – normal saline, T3 – triiodothyronine.

SUPPLEMENTAL FIGURES

Supplemental Figure 1

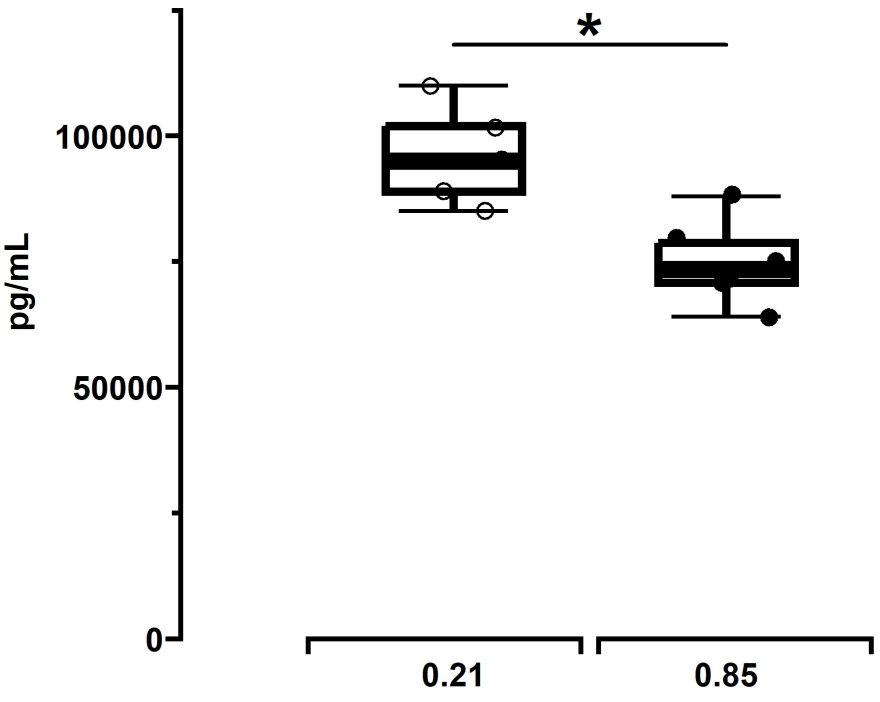

Supplemental Figure 2

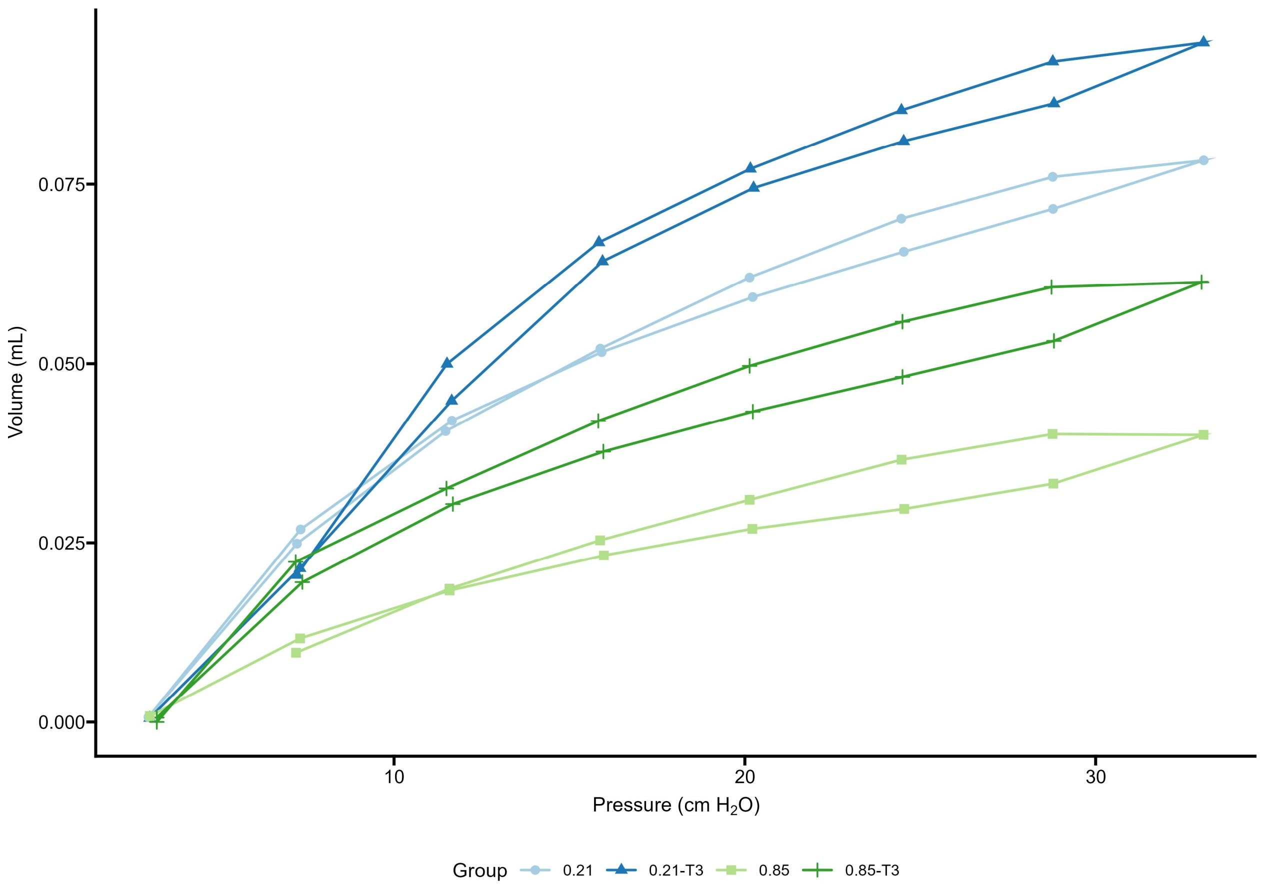

Supplemental Figure 3

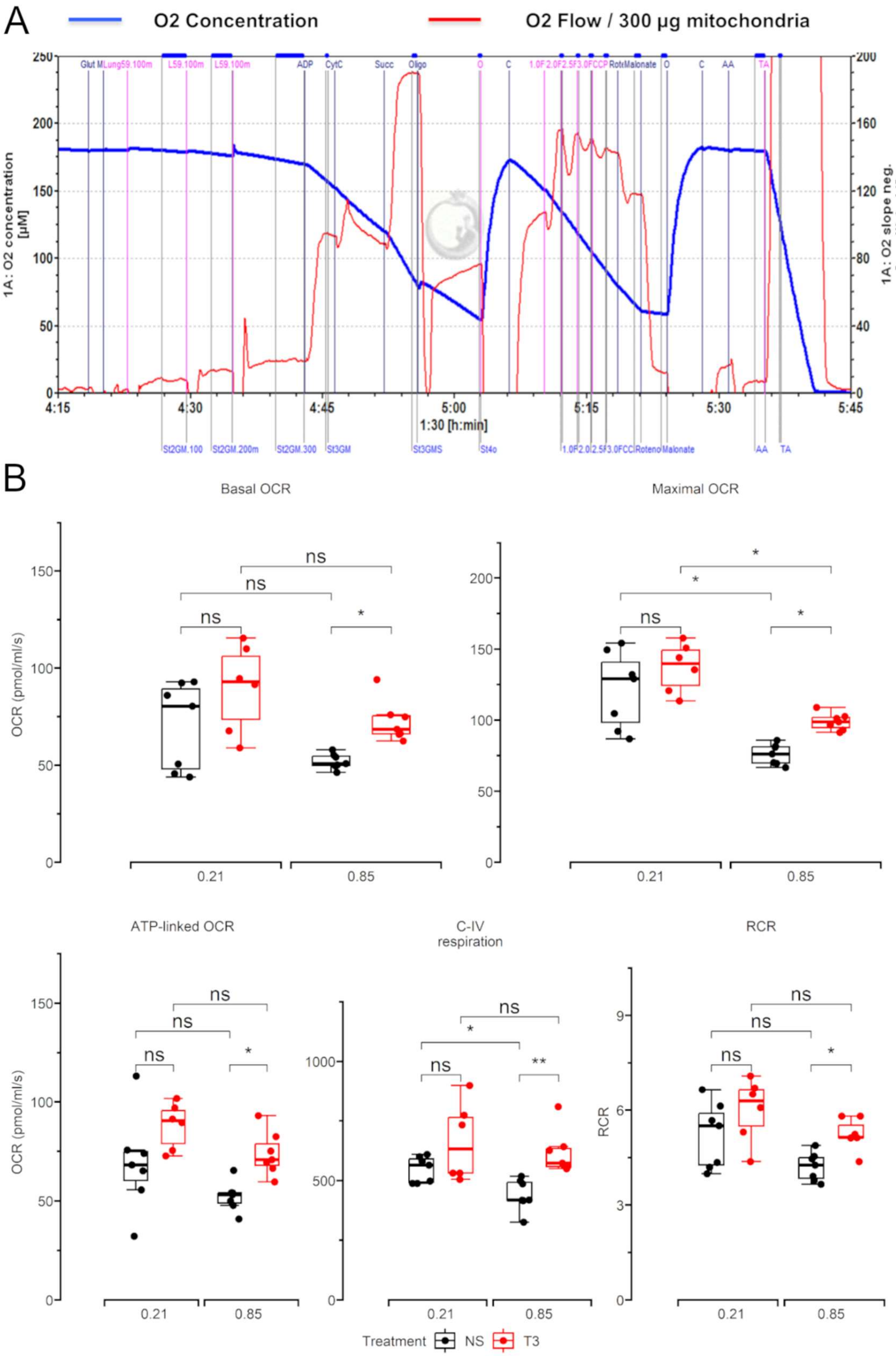

Supplemental Figure 4

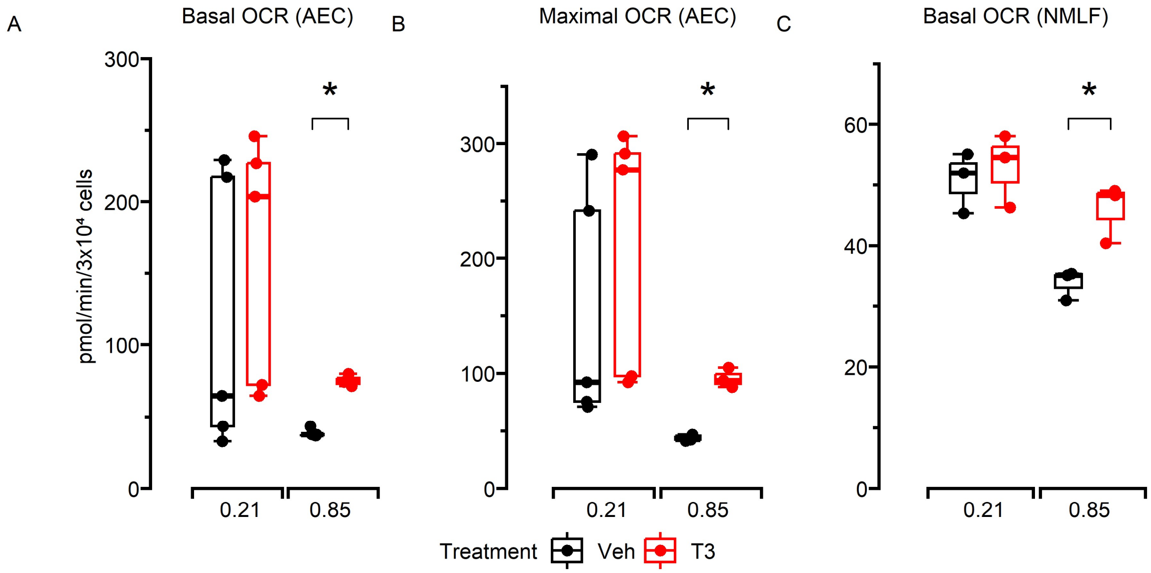

Supplemental Figure 5

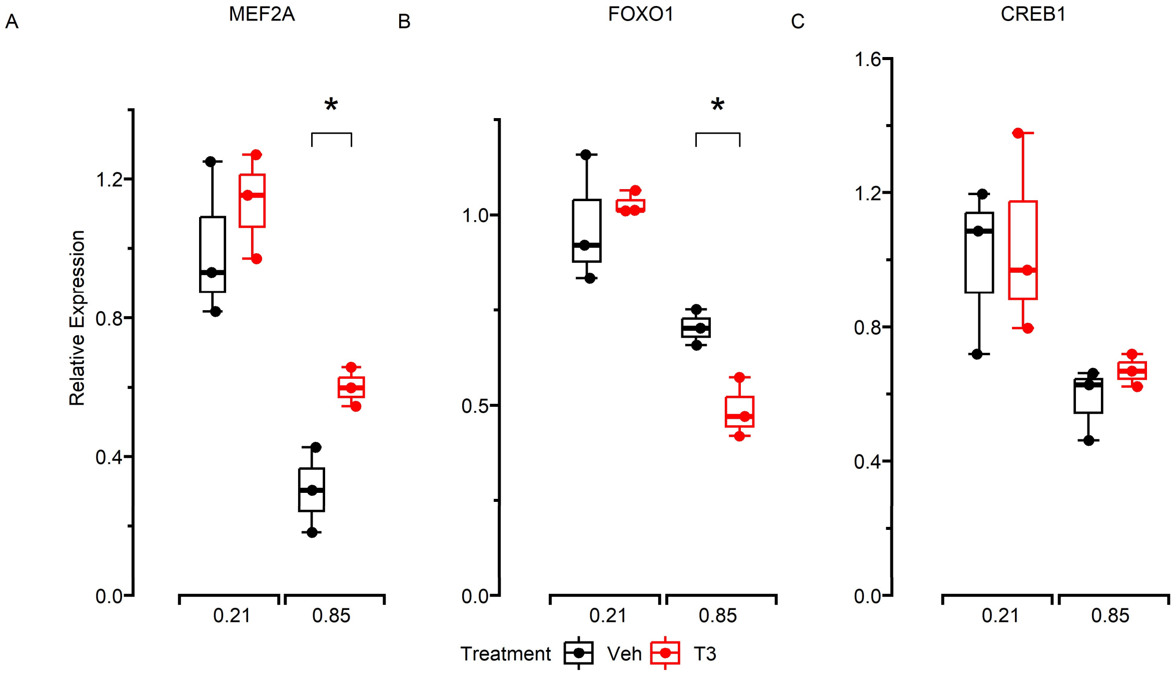

Supplemental Figure 6

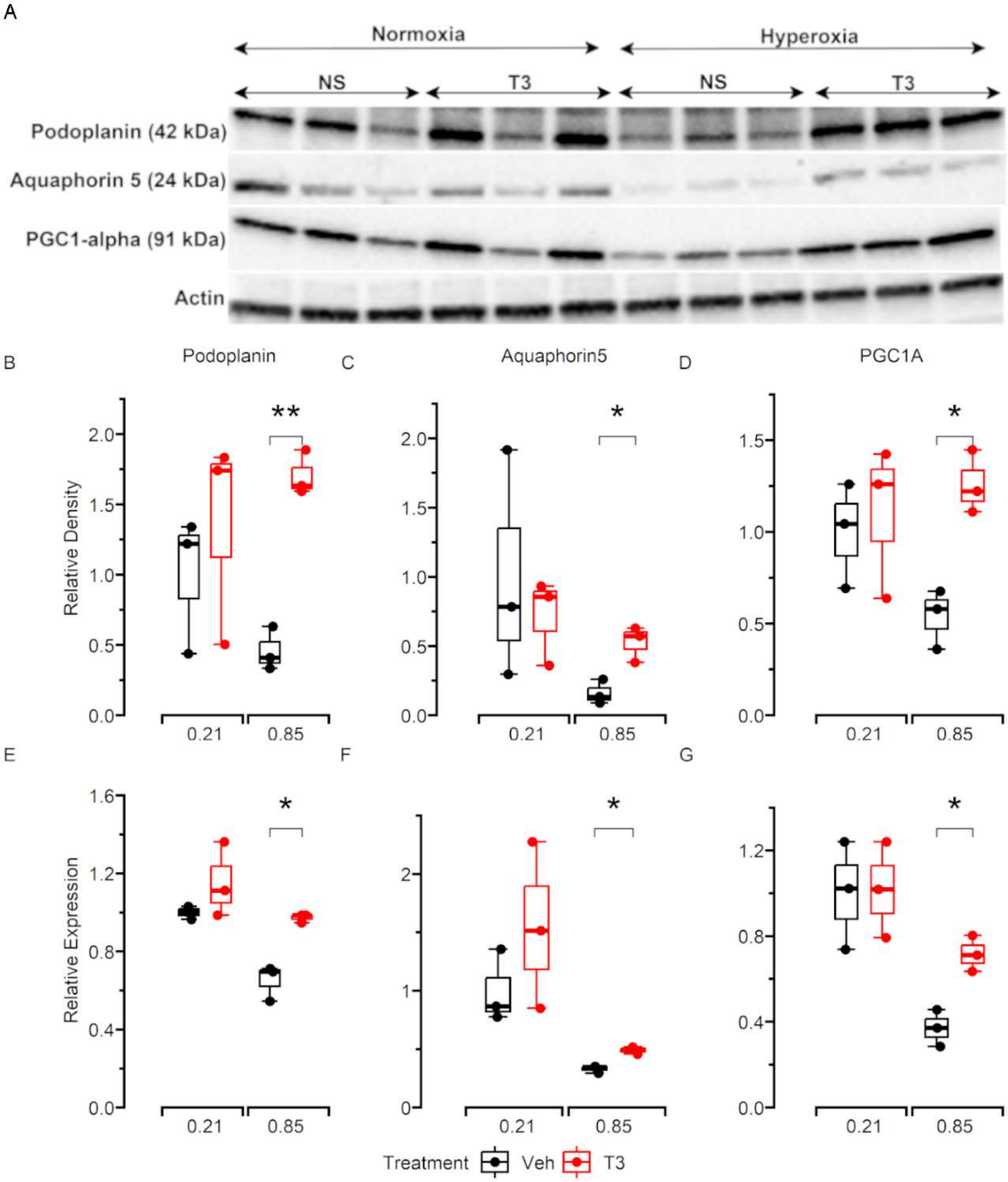

Supplemental Figure 7

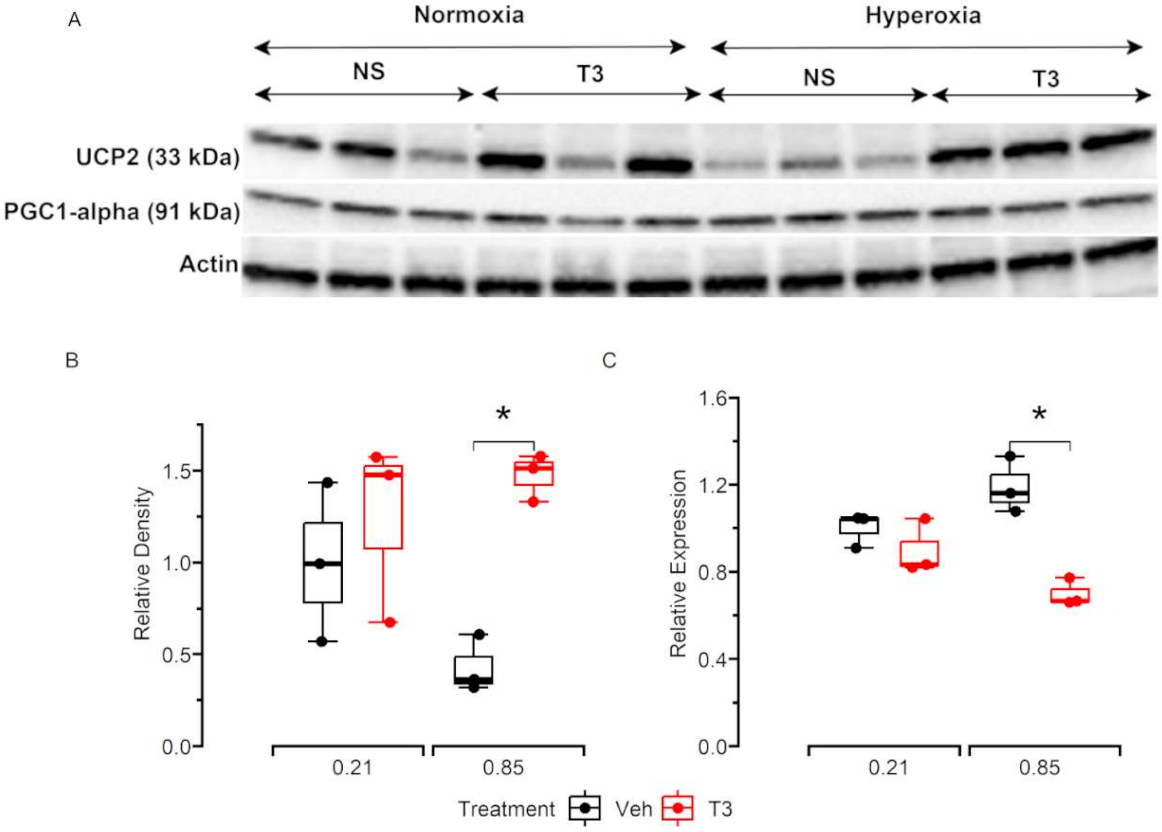

Supplemental Figure 8

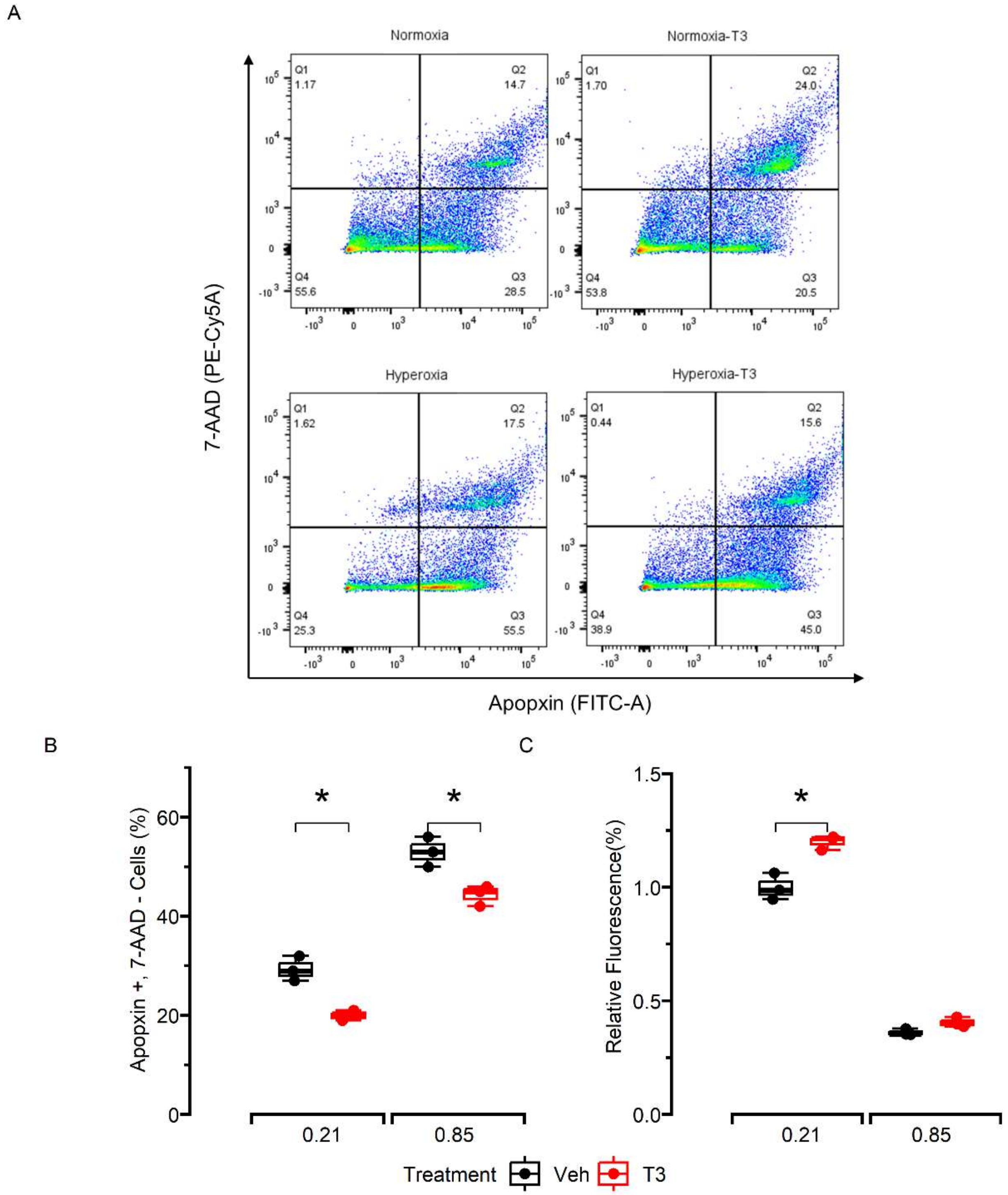

Supplemental Figure 9

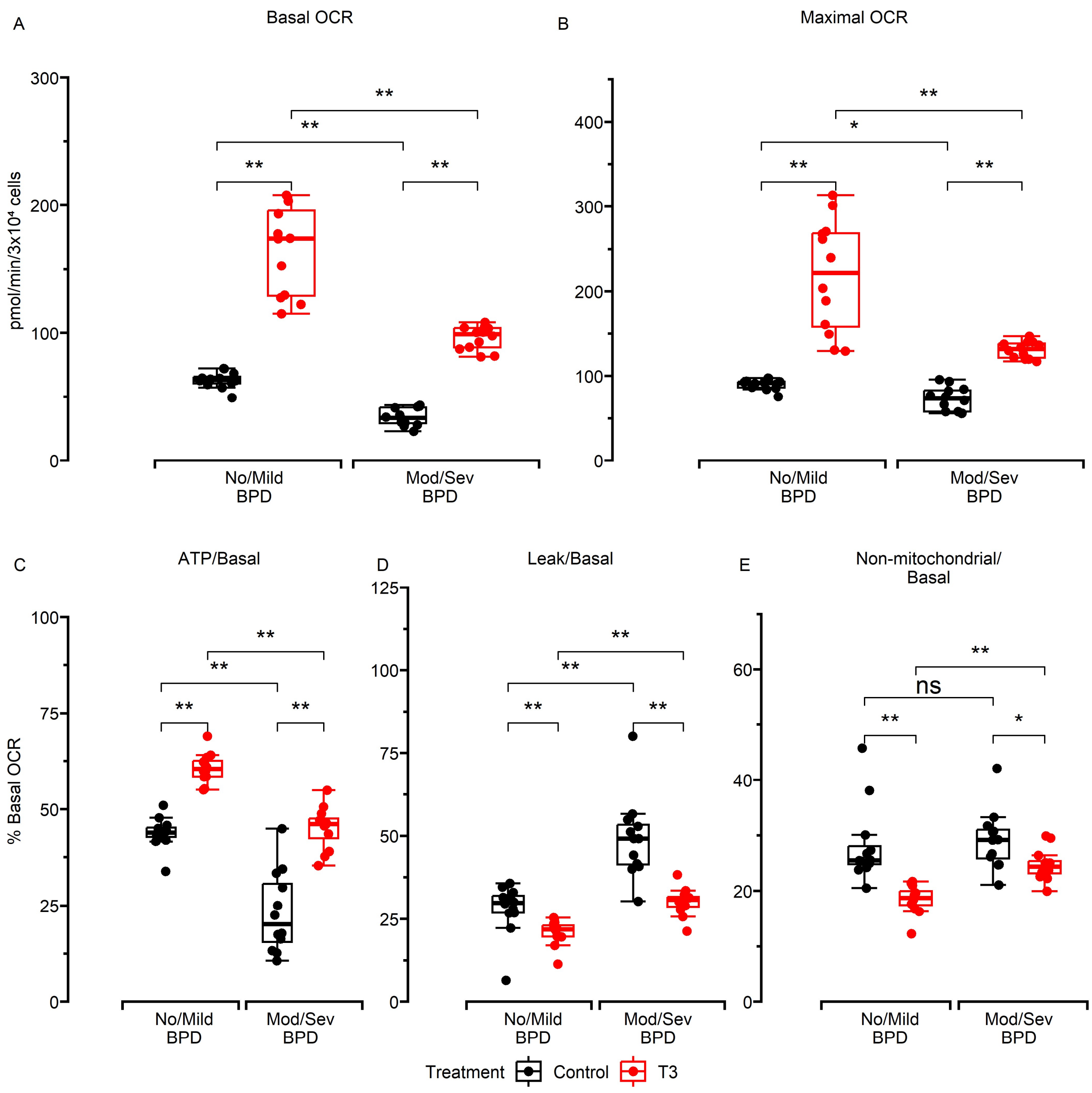

Supplemental Figure 10

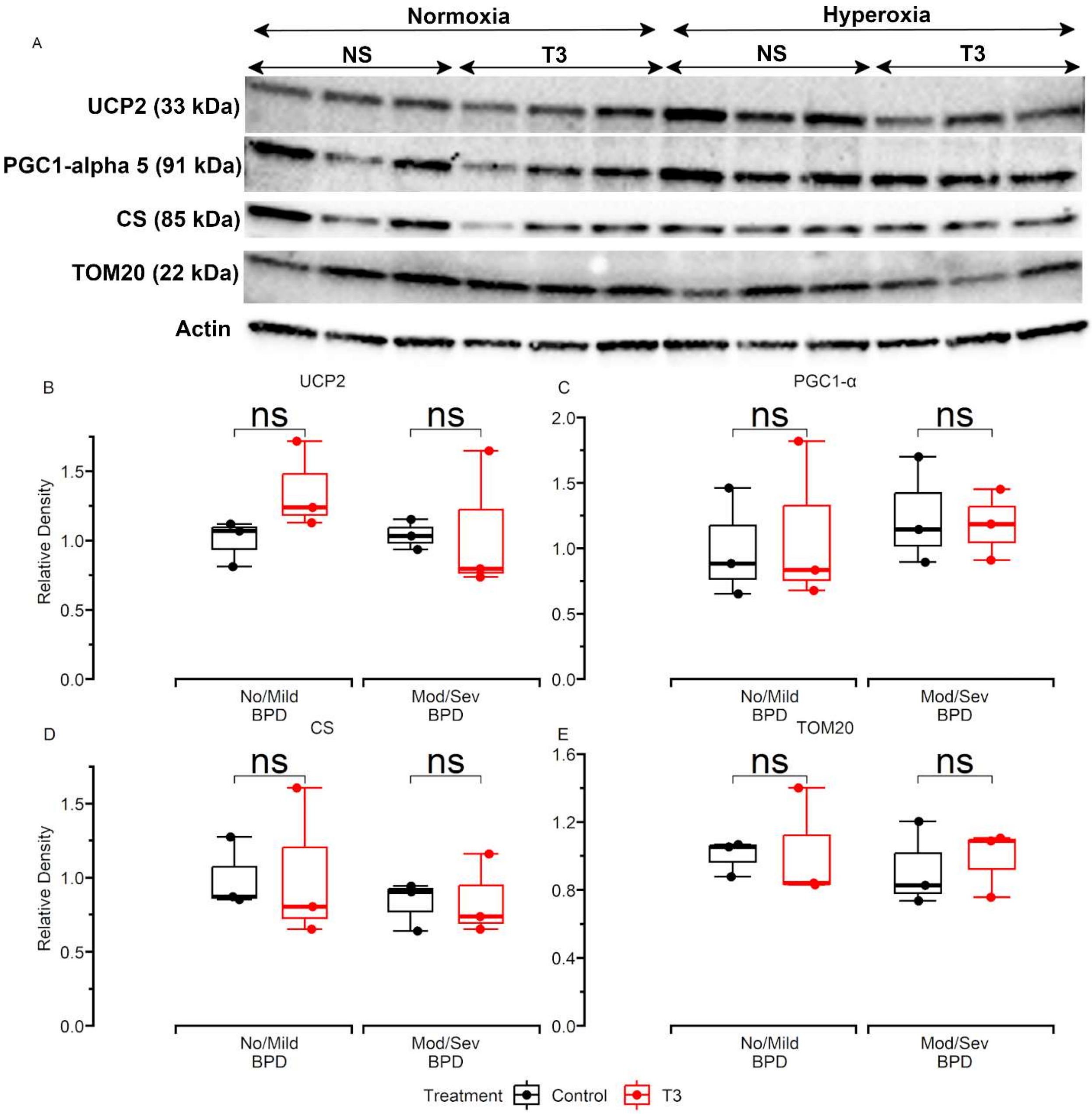

Supplement: Supplemental data [file jciinsight-8-160697-s032.pdf]
